# AARS2‐SIRT5‐Driven DLD K445 Lactylation Promotes Cuproptosis Resistance in Glioblastoma Stem Cells

**DOI:** 10.1002/advs.78046

**Published:** 2026-09-29

**Authors:** Mingtian Ding, Haibiao Xu, Cen Liu, Yangyin Ding, Shuai Wang, Jun Yin, Tao Fu, Xiao Jiang, Yuning Chen, Hui Luo, Rui Li, Zhengxin Chen, Huibo Wang

**Affiliations:** ^1^ Department of Neurosurgery The First Affiliated Hospital of Nanjing Medical University Nanjing China; ^2^ Department of Hematology The First Affiliated Hospital of Nanjing Medical University Nanjing China; ^3^ Institute for Brain Tumors Jiangsu Key Laboratory of Cancer Biomarkers Prevention and Treatment Collaborative Innovation Center for Cancer Personalized Medicine Nanjing Medical University Nanjing China

**Keywords:** cuproptosis, glioblastoma stem cells, lactylation

## Abstract

Therapy‐resistant glioblastoma stem cells (GSCs) drive tumor progression and recurrence in glioblastoma (GBM). Cuproptosis is a copper‐dependent form of regulated cell death triggered by aggregation of lipoylated mitochondrial proteins, but the mechanisms underlying cuproptosis resistance in GSCs remain unclear. Lactylome profiling identifies dihydrolipoamide dehydrogenase (DLD) as a key lactylation target regulating cuproptosis. Mechanistically, lactate‐dependent alanyl‐tRNA synthetase 2 (AARS2) catalyzes DLD K445 lactylation, while SIRT5 reverses this modification via NAD^+^‐dependent delactylation. DLD K445 lactylation does not disrupt pyruvate dehydrogenase (PDH) complex assembly or DLD stability, but suppresses PDH activity and DLAT lipoylation, limiting copper‐triggered DLAT aggregation and cuproptosis. Genetic disruption of DLD K445 lactylation restores DLAT lipoylation and sensitizes GSCs to elesclomol‐copper (ES‐Cu). Inhibiting lactate metabolism, activating SIRT5, or a cell‐penetrating peptide targeting DLD K445 all boost ES‐Cu efficacy in patient‐derived glioblastoma organoids and intracranial xenografts. Our work establishes DLD K445 lactylation as a metabolic driver of cuproptosis resistance and validates the AARS2–DLD–SIRT5 axis as a druggable therapeutic target for GBM.

## Graphical abstract

Lactylation of DLD at K445 by AARS2 suppresses PDH complex activity and reduces DLAT lipoylation, thereby conferring cuproptosis resistance in glioblastoma stem cells. SIRT5‐mediated delactylation restores PDH function and enhances cuproptosis sensitivity. Targeting DLD K445 lactylation with a specific peptide improves therapeutic responses in glioblastoma models.

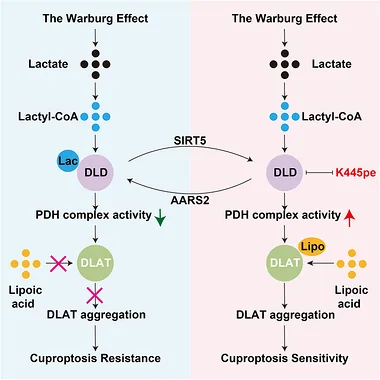

## 1 Introduction

Glioblastoma (GBM) remains the most aggressive primary malignancy of the central nervous system and is characterized by nearly universal recurrence despite maximal surgical resection, radiotherapy, and chemotherapy [1]. Mounting evidence suggests that tumor recurrence is driven by profound intratumoral heterogeneity, adaptive lineage plasticity, and the persistence of glioblastoma stem cells (GSCs) [2]. These cells feature self‐renewal, tumorigenicity, and drug stress tolerance, and remodel metabolism upon microenvironmental stimuli. In glioblastoma, mesenchymal malignant cell states correlate with invasion, inflammation, treatment resistance, and unfavorable prognosis, suggesting that this cellular state may represent a privileged niche for stress‐adaptive survival mechanisms [3].

Recent studies have identified cuproptosis as a distinct form of copper‐dependent regulated cell death. Unlike apoptosis, ferroptosis, or necroptosis, cuproptosis is intrinsically linked to mitochondrial metabolism and preferentially occurs in cells with an active tricarboxylic acid (TCA) cycle [4, 5]. Mechanistically, copper directly binds lipoylated mitochondrial proteins, particularly dihydrolipoamide S‐acetyltransferase (DLAT), triggering toxic protein aggregation, proteotoxic stress, and ultimately cell death. Elevated copper levels have been reported in several solid tumors and are associated with tumor progression [6, 7, 8]. In glioblastoma, emerging evidence suggests that dysregulated copper homeostasis may represent a therapeutic vulnerability [9]. Accordingly, copper ionophores such as elesclomol‐copper (ES‐Cu), which elevate intracellular copper levels and induce cuproptosis, have attracted increasing interest as potential therapeutic agents for GBM.

However, susceptibility to cuproptosis is not determined solely by copper availability. The abundance of lipoylated mitochondrial proteins and the activity of the pyruvate dehydrogenase (PDH) complex are key determinants of cuproptosis sensitivity, whereas broader metabolic states may further shape cellular responses to copper stress [4]. GSCs exhibit remarkable metabolic plasticity and readily transition between mitochondrial and glycolytic states [2]. Within the lactate‐rich tumor microenvironment, metabolic adaptation driven by lactate accumulation may reshape mitochondrial function and cellular metabolic dependencies [10]. Whether lactate‐driven metabolic reprogramming shapes cuproptosis susceptibility in GSCs remains unknown.

In 2019, Zhang et al. reported that lactate functions not only as a metabolic intermediate but also as a signaling molecule that drives lysine lactylation, a post‐translational modification linking cellular metabolism to gene regulation [11]. Histone lysine lactylation has since been shown to regulate gene transcription through epigenetic mechanisms [12, 13, 14, 15, 16, 17]. More recently, lactylation of non‐histone proteins has been recognized as an additional layer of metabolic control, influencing cellular adaptation and disease progression [18, 19, 20]. These observations raise the possibility that lactate metabolism may influence cuproptosis through mechanisms beyond global metabolic remodeling. Because cuproptosis is tightly coupled to mitochondrial metabolism and lipoylated protein homeostasis, mitochondrial proteins involved in these processes may represent functionally important targets of lactylation. However, whether protein lactylation directly regulates the mitochondrial machinery governing cuproptosis remains unknown.

We report dihydrolipoamide dehydrogenase (DLD) K445 lactylation as a metabolic driver of cuproptosis resistance in mesenchymal GSCs upon lactate exposure. Lactate‐induced, alanyl‐tRNA synthetase 2 (AARS2)‐dependent DLD K445 lactylation inhibits DLAT lipoylation and copper‐triggered DLAT aggregation to block cuproptosis, whereas SIRT5 removes this lactylation to recover PDH activity, DLAT lipoylation, and cuproptosis sensitivity. We designed a cell‐penetrating peptide targeting the DLD K445 lactylation motif; it reduces DLD lactylation and sensitizes GSCs to cuproptosis in organoid and PDX models. Collectively, DLD K445 lactylation represents a novel metabolic checkpoint controlling cuproptosis resistance, providing a druggable approach to ablate adaptive survival in glioblastoma.

## 2 Result

### 2.1 Lactate Metabolism is Associated With Cuproptosis Resistance in Mesenchymal‐Like GSCs

We first examined whether copper accumulation is associated with glioblastoma progression and cuproptosis‐related phenotypes. Copper concentrations were significantly elevated in GBM tissues compared with paired normal brain tissues and further increased in higher‐grade gliomas (Figure 1A,B). In line with the tissue distribution pattern, patients harboring tumors with high copper levels exhibited significantly shorter overall survival than those with low copper levels (Figure 1C), suggesting that copper accumulation accompanies aggressive glioblastoma progression.

**FIGURE 1:**
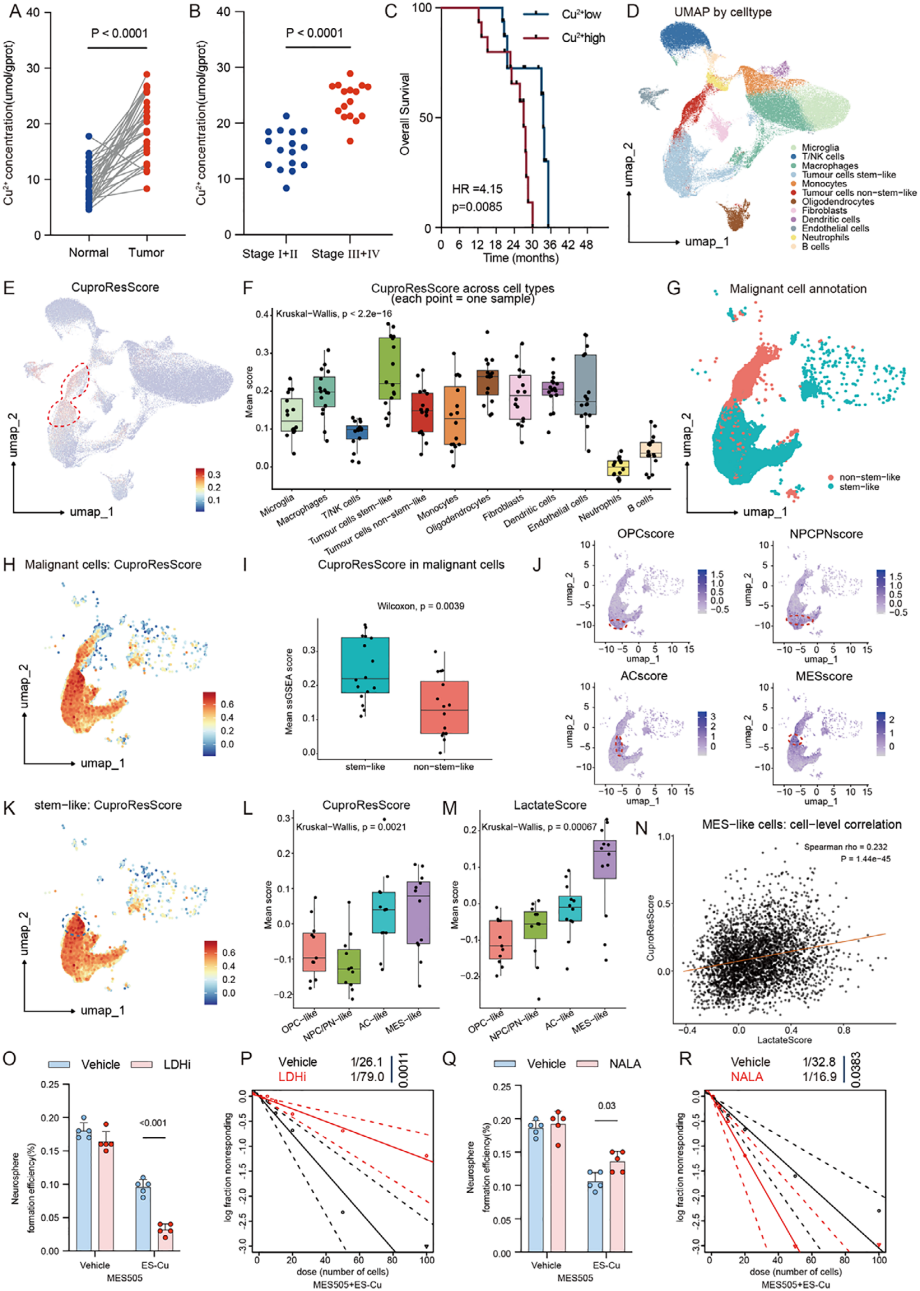
Lactate metabolism is associated with cuproptosis resistance in mesenchymal‐like glioblastoma stem cells. (A) Cu^2^^+^ concentrations in paired normal brain and glioblastoma tissues. (B) Cu^2^^+^ concentrations in glioblastoma samples stratified by WHO grade. (C) Kaplan–Meier analysis of overall survival in patients with high or low intratumoral Cu^2^^+^ levels. (D) UMAP visualization of glioblastoma single‐cell transcriptomes colored by annotated cell populations. (E, F) Distribution and quantification of CuproResScore across major cellular populations. (G–I) Identification of stem‐like and non‐stem‐like malignant cells and analysis of CuproResScore in these populations. (J) Cell‐state scoring of OPC‐like, NPC/PN‐like, AC‐like, and MES‐like stem‐like malignant cells. (K) UMAP projection of CuproResScore within stem‐like malignant cells. (L, M) Comparison of CuproResScore and LactateScore among distinct stem‐like malignant cell states. (N) Cell‐level correlation between LactateScore and CuproResScore in MES‐like malignant cells. (O, P) Effects of LDH inhibition on neurosphere formation and stem‐cell frequency in MES505 cells in the presence or absence of ES‐Cu. (Q, R) Effects of NALA supplementation on neurosphere formation and stem‐cell frequency in MES505 cells in the presence or absence of ES‐Cu. Data are presented as mean ± SD unless otherwise indicated. n = 3 biologically independent samples for O and Q. For single‐cell analyses, each point represents one independent sample. P values were determined by paired two‐sided t‐test for A, unpaired two‐sided t‐test for B and I, Kruskal–Wallis test for F, L and M, Spearman correlation test for N, ELDA chi‐square test for P and R, and log‐rank (Mantel–Cox) test for C. NS, not significant.

To determine whether cuproptosis‐resistant states are preferentially enriched within specific cellular compartments of the GBM ecosystem, we performed single‐cell transcriptomic analysis. Unsupervised clustering identified malignant tumor cells together with major non‐malignant populations, including microglia, macrophages, monocytes, T/NK cells, oligodendrocytes, fibroblasts, dendritic cells, endothelial cells, neutrophils, and B cells (Figure 1D and Figure S1). Mapping of CuproResScore across the cellular landscape revealed a non‐random distribution, with enrichment in discrete malignant‐cell regions rather than across the entire tumor microenvironment (Figure 1E). Quantitative analysis further demonstrated that stem‐like malignant cells exhibited the highest CuproResScore among major cellular populations and significantly higher scores than non‐stem‐like malignant cells (Figure 1F–I), indicating that cuproptosis resistance is preferentially associated with stem‐like tumor states.

Given the established cellular plasticity of GBM stem cells, we next examined whether cuproptosis resistance varied across distinct malignant‐cell states. Classification of stem‐like malignant cells into OPC‐like, NPC/PN‐like, AC‐like, and MES‐like programs revealed marked state‐dependent differences. MES‐like cells displayed the highest CuproResScore among all stem‐like states and simultaneously exhibited the strongest lactate‐associated metabolic signature, as reflected by elevated LactateScore (Figure 1J–M). Single‐cell analysis demonstrated a significant positive correlation between LactateScore and CuproResScore within MES‐like cells (Figure 1N), supporting a close relationship between lactate‐associated metabolic activity and acquisition of a cuproptosis‐resistant phenotype.

Several orthogonal analyses further supported this association. LactateScore was significantly elevated in malignant cells relative to non‐malignant cells, positively correlated with CuproResScore at the patient level, and stratified tumors with increased CuproResScore in both the CPTAC and TCGA cohorts (Figure S2A–D). Immunofluorescence analyses of human GBM specimens demonstrated that ALDH1A3‐positive MES‐like malignant cells were characterized by abundant LDHA expression but relatively low DLAT expression (Figure S2E,F), consistent with enhanced lactate metabolism and reduced abundance of a key cuproptosis effector protein. Collectively, the data identify a metabolic state characterized by elevated lactate metabolism and enhanced cuproptosis resistance that is preferentially enriched within mesenchymal‐like GSCs.

To interrogate whether lactate metabolism governs ES‐Cu toxicity in mesenchymal GSCs, we first measured ES‐Cu sensitivity across a panel of glioma cell lines and patient‐derived GSCs (Figure S3A). ES‐Cu exhibited potent cytotoxicity in all models, with MES505 and MES21 mesenchymal GSCs selected for mechanistic follow‐up.

Pharmacological LDH inhibition simultaneously reduced intracellular lactate and global pan‐lactylation (Figure S3B–D), and exacerbated ES‐Cu‐induced cell death and suppression of self‐renewal in MES‐GSCs (Figure S3E–H).

To separate the contribution of protein lactylation from bulk lactate pools, we treated cells with NALA, a lactylation precursor that does not raise cellular lactate concentrations. NALA markedly elevated pan‐lactylation without altering intracellular lactate levels (Figure S3I–K), and attenuated ES‐Cu‐mediated growth suppression while preserving stem‐like properties in MES505/MES21 cells (Figure S3L–O). These reciprocal phenotypes decouple protein lactylation from total lactate abundance, supporting lysine lactylation as a functional mediator of resistance to ES‐Cu‐induced cytotoxicity.

Genetic LDHA knockdown recapitulated the sensitizing effect of LDH inhibition: LDHA depletion lowered lactate and pan‐lactylation, and increased MES‐GSC sensitivity to ES‐Cu treatment (Figure S3P–T).

Together, these gain‐ and loss‐of‐function pharmacological and genetic assays establish protein lactylation as a key determinant of ES‐Cu responsiveness in mesenchymal glioblastoma stem cells. Suppressing lactate/LDHA production reduces lactylation and enhances ES‐Cu activity, whereas NALA‐driven lactylation enhancement—independent of lactate concentration—promotes resistance to ES‐Cu anti‐GSC effects.

### 2.2 DLD is Lactylated by AARS2

To systematically identify lactylation events potentially linked to cuproptosis regulation in GBM, we performed 4D label‐free quantitative lactylome profiling. This analysis identified 3 361 lysine lactylation sites across 1 473 proteins (Table S1). Among proteins with established relevance to cuproptosis‐related pathways, DLD and GLS emerged as prominent candidates. Immunoprecipitation followed by pan‐lactylation immunoblotting revealed robust lactylation of DLD, whereas GLS exhibited little detectable lactylation under identical conditions (Figure 2A), suggesting that lactylation may selectively target specific components of cuproptosis‐associated metabolic machinery.

**FIGURE 2:**
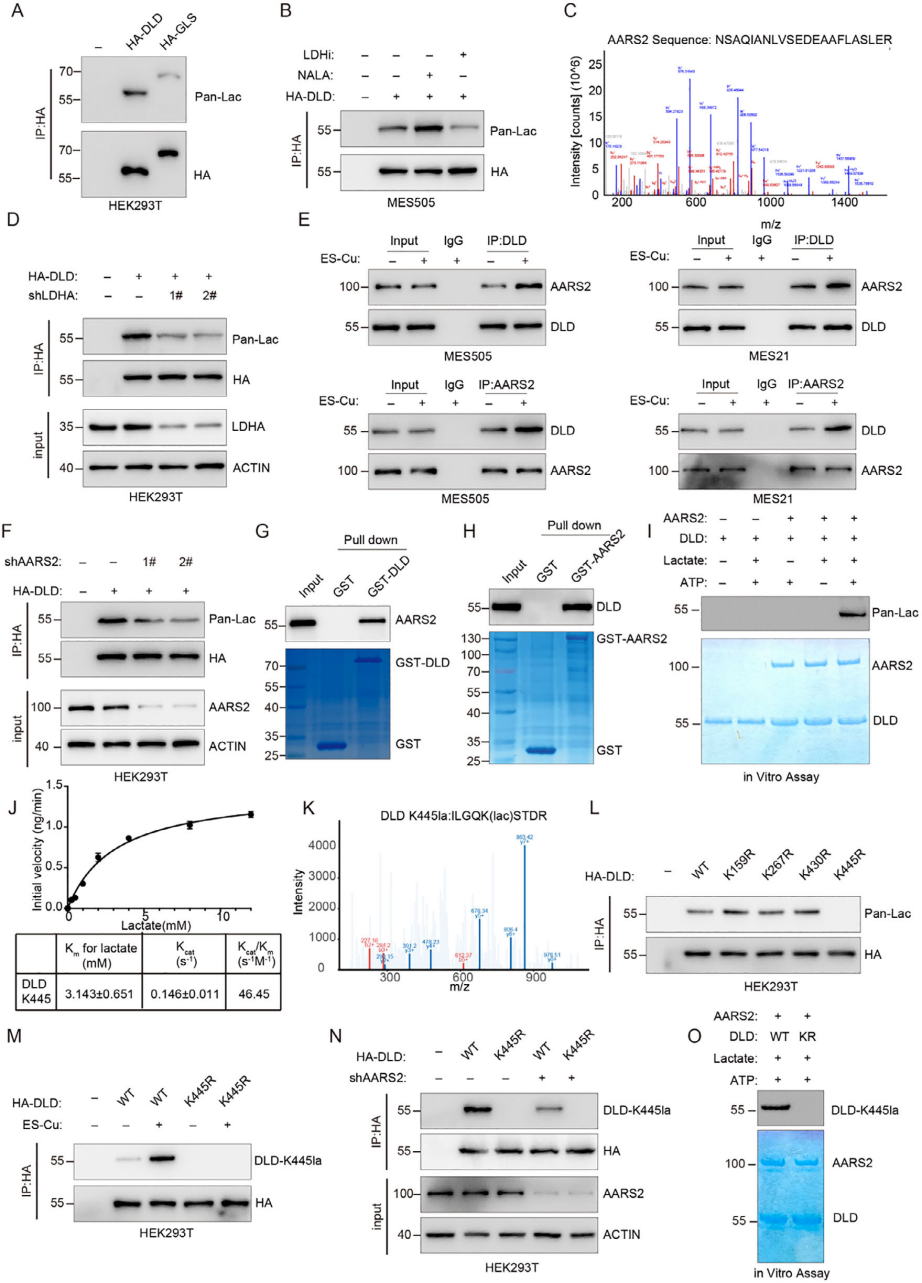
DLD is lactylated at K445 by AARS2. (A) Pan‐lysine lactylation detected in HA‐DLD and HA‐GLS immunoprecipitates from HEK293T cells. (B) DLD lactylation in MES505 cells following treatment with an LDH inhibitor (LDHi) or NALA. (C) Representative MS/MS spectrum of the AARS2‐derived peptide NSAQIANLVSEDEAAFLASLER identified in DLD‐associated protein complexes. (D) Effects of LDHA knockdown on DLD lactylation in HEK293T cells. (E) Reciprocal co‐immunoprecipitation analyses of endogenous DLD and AARS2 in MES505 and MES21 cells in the presence or absence of ES‐Cu. (F) Effects of AARS2 depletion on DLD lactylation in HEK293T cells. (G, H) GST pull‐down assays demonstrating direct binding between DLD and AARS2. (I) In vitro lactylation assay showing that AARS2 mediates DLD lactylation in the presence of lactate and ATP. Recombinant DLD was incubated with AARS2 under the indicated conditions, and DLD‐K445 lactylation was detected by immunoblotting. (J) Michaelis–Menten kinetic analysis of AARS2‐mediated DLD K445 lactylation using increasing concentrations of lactate. The calculated kinetic parameters (Km, Kcat, and Kcat/Km) are shown. (K) Representative MS/MS spectrum confirming the DLD K445 lactylation site (K445). (L) Validation of DLD lactylation sites by mutagenesis. HA‐DLD WT or indicated lysine‐to‐arginine mutants were immunoprecipitated and analyzed for pan‐lactylation levels. (M) ES‐Cu treatment promotes DLD K445 lactylation in HA‐DLD WT but not in the K445R mutant protein. (N) AARS2 knockdown reduces DLD K445 lactylation in HA‐DLD WT but not in the K445R mutant protein. (O) In vitro lactylation assay demonstrating that AARS2 selectively catalyzes lactylation of WT DLD but not K445R mutant DLD in the presence of lactate and ATP. Original unprocessed blot images are provided in Figure S12A.

We next examined whether DLD lactylation is responsive to lactate metabolism. In MES505 cells, supplementation with NALA markedly increased DLD lactylation, whereas pharmacological inhibition of LDH reduced lactylation levels (Figure 2B). To identify the enzyme responsible for DLD lactylation, we analyzed DLD‐associated proteins by mass spectrometry and detected peptides derived from AARS2 among DLD‐interacting proteins (Figure 2C and Table S2). Genetic depletion of LDHA similarly diminished DLD lactylation (Figure 2D). Importantly, treatment with NALA did not significantly alter the expression of core cuproptosis regulators, including FDX1, LIAS, LIPT1, DLD and DLAT, whereas exogenous L‐lactate altered the expression of several of these proteins (Figure S4A). These findings suggest that NALA‐induced lactylation regulates cuproptosis predominantly through post‐translational mechanisms, while exogenous lactate may additionally affect cuproptosis‐related protein expression. Pharmacological inhibition of MCT1/4 increased intracellular lactate accumulation and enhanced DLD lactylation, whereas inhibition of GLO1 exerted minimal effects (Figure S4B–D), arguing against a major contribution of non‐enzymatic glyoxalase‐dependent lactylation in this setting.

AARS2 has recently been reported to function as a lactate sensor and lactyltransferase^22^. Consistent with this function, reciprocal co‐immunoprecipitation experiments confirmed endogenous interaction between DLD and AARS2 in MES505 and MES21 cells, and this interaction was further enhanced following ES‐Cu treatment (Figure 2E), suggesting that copper stress promotes assembly of the DLD–AARS2 complex. Knockdown of AARS2 markedly reduced DLD lactylation (Figure 2F). Direct interaction between DLD and AARS2 was further confirmed by reciprocal GST pull‐down assays using purified proteins (Figure 2G,H). In vitro lactylation assays demonstrated that purified AARS2 directly catalyzed DLD lactylation in a time‐dependent and lactate/ATP‐dependent manner (Figure 2I and Figure S4E). Kinetic analysis of this enzymatic reaction revealed measurable catalytic parameters for AARS2‐mediated DLD K445 lactylation, with a calculated Km of 3.143 ± 0.651 mm for lactate and a catalytic efficiency (Kcat/Km) of 46.45 s^−^^1^M^−^^1^ (Figure 2J). These results provide direct biochemical evidence that AARS2 functions as a writer enzyme responsible for DLD lactylation.

### 2.3 DLD is Lactylated at K445

DLD is an evolutionarily conserved protein composed of four structural domains: the FAD‐binding domain, the NAD‐binding domain, the central domain, and the interface domain [21]. Analysis of the proteomic dataset identified four potential lactylation sites on DLD (K159, K267, K430, and K445) (Figure 2K and Figure S4H–J). Systematic lysine‐to‐arginine mutagenesis revealed that substitution of K445 produced the most pronounced reduction in DLD lactylation, whereas mutation of other candidate residues had comparatively modest effects (Figure 2L), identifying K445 as the dominant lactylation site. ES‐Cu treatment markedly increased lactylation of DLD WT but failed to induce comparable lactylation of the K445R mutant (Figure S4F). Sequence alignment showed that K445 is evolutionarily conserved across vertebrate species (Figure S4G).

To validate this modification experimentally, we generated a site‐specific antibody recognizing lactylated DLD at K445 (DLD‐K445la). Dot‐blot assays confirmed its specificity toward the lactylated peptide (Figure S4K). Using this antibody, ES–Cu treatment was found to markedly increase DLD K445 lactylation, whereas mutation of K445 to arginine abolished the signal (Figure 2M). To further determine whether AARS2 directly controls DLD K445 lactylation, we examined the effect of AARS2 depletion in cells expressing HA‐DLD WT or the K445R mutant. AARS2 knockdown markedly reduced DLD K445 lactylation in WT DLD‐expressing cells, whereas the K445R mutant showed minimal lactylation that was not further affected by AARS2 depletion (Figure 2N). Moreover, in vitro lactylation assays using purified proteins demonstrated that AARS2 selectively catalyzed lactylation of WT DLD but not the K445R mutant in the presence of lactate and ATP (Figure 2O). These findings establish K445 as the major substrate site for AARS2‐mediated DLD lactylation.

Because K445 has previously been reported as a potential ubiquitination site [22], we next investigated whether lactylation at this site influences DLD stability. However, neither ubiquitination assays nor cycloheximide‐chase analyses revealed appreciable differences between WT and K445R DLD proteins (Figure S4L,M). These observations indicate that K445 lactylation does not regulate DLD protein abundance or turnover, suggesting instead that this modification may influence the biochemical activity or cuproptosis‐related function of DLD. Accordingly, we next investigated how DLD K445 lactylation affects mitochondrial metabolism and cuproptosis susceptibility.

### 2.4 DLD Lactylation Impairs PDH Enzymatic Activity

To determine whether DLD K445 lactylation directly affects the intrinsic enzymatic activity of DLD, we performed an in vitro enzymatic assay using purified recombinant DLD proteins. Compared with unmodified WT DLD, lactylated WT DLD exhibited reduced catalytic activity, whereas the K445R mutant largely preserved DLD activity (Figure 3A). These results indicate that K445 lactylation directly suppresses DLD enzymatic function.DLD functions as the E3 component of several mitochondrial enzyme complexes, including the pyruvate dehydrogenase complex (PDC), α‐ketoglutarate dehydrogenase complex, branched‐chain α‐keto acid dehydrogenase complex, and the glycine cleavage system [23]. Because the PDC plays a critical role in cuproptosis regulation, we next examined whether DLD lactylation influences its function.

**FIGURE 3:**
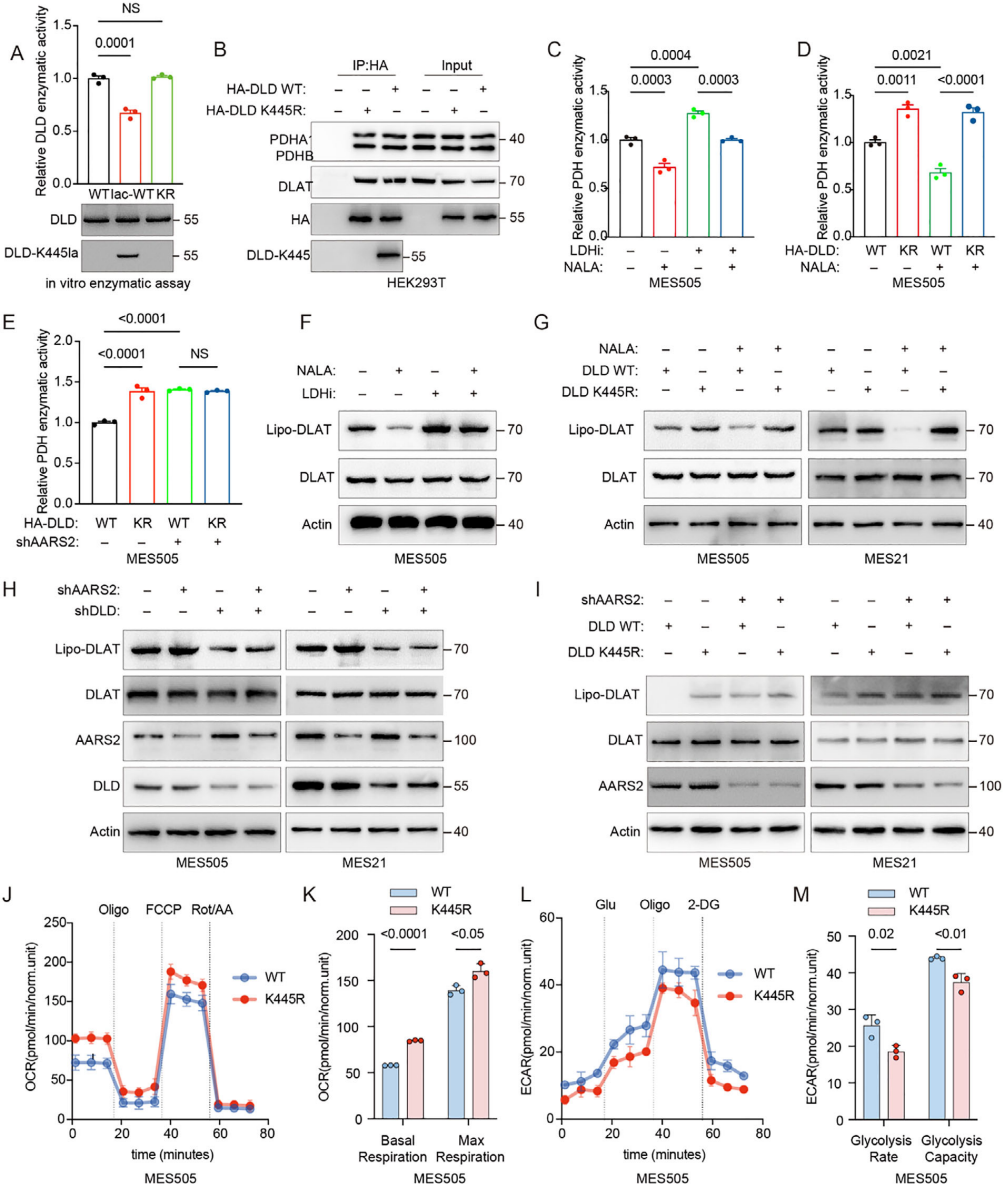
DLD K445 lactylation suppresses PDH activity and DLAT lipoylation without disrupting PDH complex assembly. (A) In vitro enzymatic assay of recombinant DLD proteins. The enzymatic activity of WT DLD, lactylated WT DLD (lac‐WT), and K445R mutant DLD was measured. DLD‐K445 lactylation was validated by immunoblotting, and total DLD protein levels were shown as loading controls. (B) Co‐immunoprecipitation analysis of PDH complex components in HEK293T cells expressing HA‐DLD WT or K445R. (C) Relative PDH activity in MES505 cells treated with LDH inhibitor (LDHi) and/or NALA. (D) Relative PDH activity in MES505 cells expressing DLD WT or K445R in the presence or absence of NALA. (E) Relative PDH activity in DLD WT‐ or K445R‐expressing MES505 cells following AARS2 knockdown. (F) DLAT lipoylation in MES505 cells treated with NALA and/or LDHi. (G) DLAT lipoylation in MES505 and MES21 cells expressing DLD WT or K445R with or without NALA treatment. (H) Effects of combined AARS2 and DLD depletion on DLAT lipoylation in MES505 and MES21 cells. (I) DLAT lipoylation following re‐expression of DLD WT or K445R in AARS2‐depleted MES505 and MES21 cells. (J, K) Oxygen consumption rate (OCR) traces and quantification of basal and maximal respiration in MES505 cells expressing DLD WT or K445R. (L, M) Extracellular acidification rate (ECAR) traces and quantification of glycolytic rate and glycolytic capacity in MES505 cells expressing DLD WT or K445R. Data are presented as mean ± SD. n = 3 biologically independent samples for C–E, J, and L. P values were determined by one‐way ANOVA with Tukey's multiple‐comparison test for C–E and by two‐sided unpaired t‐test for K and M. Original unprocessed blot images are provided in Figure S12B.

To investigate the conformational impact of K445 lactylation on DLD, we conducted 100 ns molecular dynamics simulations using the truncated DLD homodimer template PDB 2F5Z (Figure S5A). Crystal Lys‐410 aligns to full‐length DLD Lys445, as the template lacks 35 N‐terminal residues cleaved during maturation.

Global RMSD and radius of gyration (Rg) curves confirmed that wild‐type and lactylated DLD both reached stable equilibrium with highly similar final values, showing intact global folding and compactness of the dimer (Figure S5B–F). Residue‐resolved RMSF detected enhanced local flexibility exclusively around the lactylated Lys410 catalytic region. Intramolecular hydrogen bonds and SASA were largely comparable between groups, with minor structural perturbations limited to the modified pocket. These simulations indicate K445 lactylation only induces localized disorder near the modification site, without global DLD unfolding. Co‐immunoprecipitation analyses demonstrated that both WT DLD and the K445R mutant retained interactions with PDHA1, PDHB, and DLAT (Figure 3B), indicating that K445 lactylation does not induce major conformational instability or structural disassembly of the PDH complex.

To examine whether K445 lactylation alters PDH enzymatic activity, we measured PDH activity after bidirectional manipulation of cellular lactylation. In MES505 cells, NALA treatment significantly reduced PDH activity, whereas LDH inhibition increased PDH activity (Figure 3C). Expression of the lactylation‐deficient K445R mutant enhanced PDH activity compared with WT DLD and largely abolished the inhibitory effect of NALA (Figure 3D). Genetic depletion of AARS2 increased PDH activity in WT DLD‐expressing cells but failed to produce additional activation in K445R‐expressing cells (Figure 3E), genetically placing DLD K445 lactylation downstream of AARS2. Similar results were observed in MES21 cells and were further supported by complementary gain‐ and loss‐of‐function analyses (Figure S6A–H).

### 2.5 DLD Lactylation Inhibits Protein Lipoylation

Lipoylated DLAT serves as the primary copper‐binding target during cuproptosis [4]. We therefore examined whether DLD lactylation influences protein lipoylation. NALA reduced DLAT lipoylation, whereas LDH inhibition increased DLAT lipoylation in MES505 cells (Figure 3F). Expression of DLD K445R restored DLAT lipoylation despite NALA treatment in both MES505 and MES21 cells (Figure 3G). Loss of DLD abolished the effects of AARS2 knockdown on DLAT lipoylation, while re‐expression of WT DLD, but not regulation through the K445 lactylation‐deficient pathway, restored responsiveness to AARS2 signaling (Figure 3H,I and Figure S6I–K). These data establish DLD as the essential downstream effector through which AARS2‐dependent lactylation controls DLAT lipoylation.

We next examined the metabolic consequences of DLD K445 lactylation. Seahorse analysis revealed that cells expressing DLD K445R displayed significantly higher basal and maximal oxygen consumption rates than WT‐expressing cells, indicating enhanced mitochondrial oxidative metabolism (Figure 3J,K). Conversely, K445R expression reduced extracellular acidification rate, glycolytic activity, and glycolytic capacity (Figure 3L,M). Similar metabolic reprogramming was observed in MES21 cells (Figure S6L–O). Collectively, the data indicate that DLD K445 lactylation suppresses PDH activity and limits DLAT lipoylation without altering PDH complex assembly, shifting cellular metabolism away from oxidative phosphorylation toward glycolysis. Such a metabolic state would reduce the pool of lipoylated mitochondrial substrates required for cuproptosis and thereby promote copper tolerance.

### 2.6 Loss of DLD K445 Lactylation Sensitizes GSCs to ES‐Cu‐Induced Cuproptosis

Because aggregation of lipoylated DLAT represents a defining biochemical hallmark of cuproptosis, we next examined whether DLD K445 lactylation regulates the execution of copper‐induced cell death. Following ES‐Cu exposure, MES505 and MES21 cells expressing the lactylation‐deficient DLD K445R mutant exhibited markedly enhanced formation of high‐molecular‐weight DLAT oligomers compared with WT DLD‐expressing cells (Figure 4A,B). Supporting these biochemical observations, immunofluorescence analyses revealed substantially increased mitochondrial DLAT puncta in K445R‐expressing cells after ES‐Cu treatment, indicative of enhanced DLAT aggregation and cuproptotic stress (Figure 4C–F). In contrast, lactate supplementation with NALA suppressed ES‐Cu‐induced DLAT aggregation, whereas LDH inhibition potentiated both DLAT puncta formation and oligomer accumulation (Figure S7A–F). These data indicate that DLD K445 lactylation restrains the aggregation of lipoylated DLAT, a critical initiating event in cuproptosis.

**FIGURE 4:**
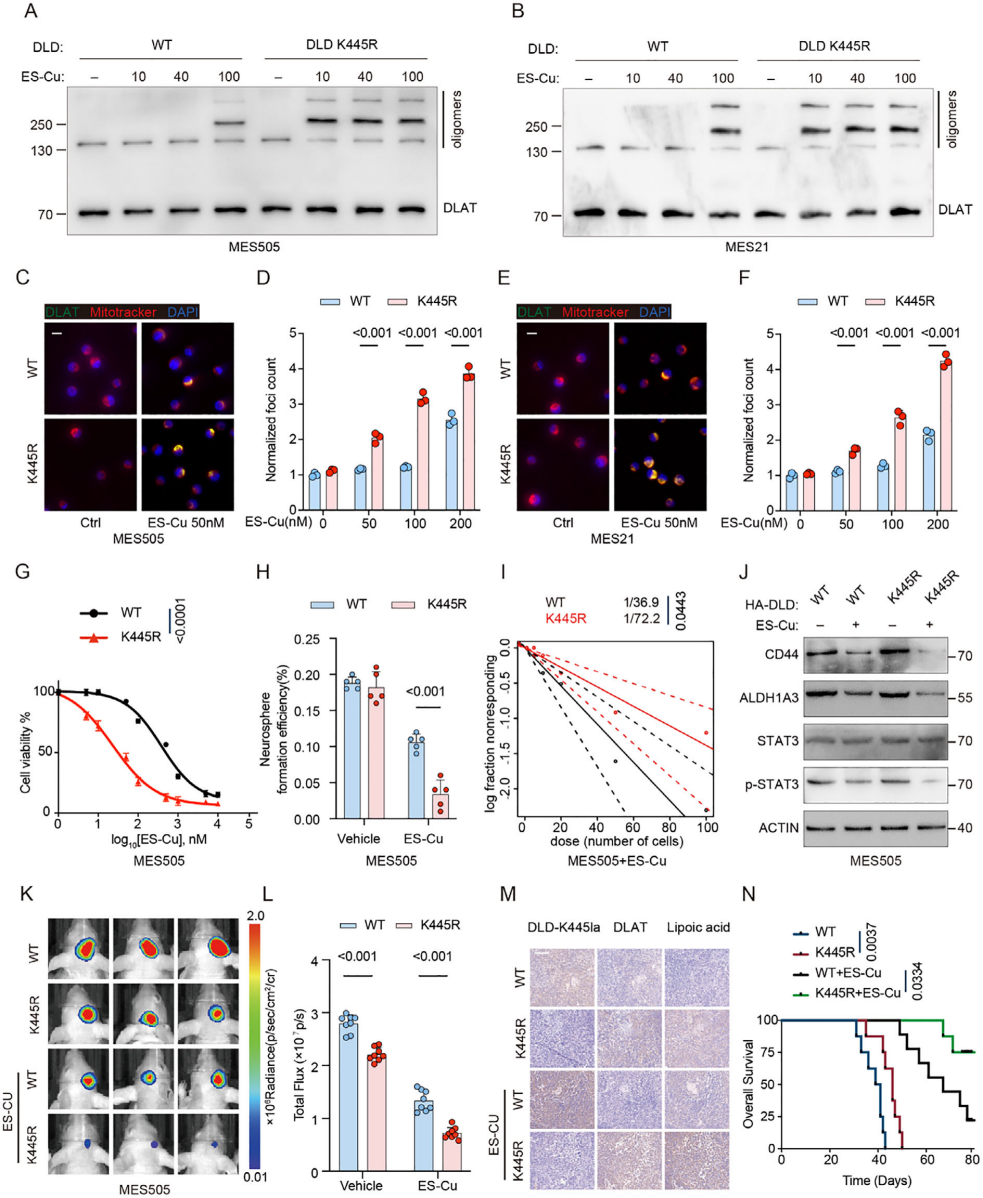
Loss of DLD K445 lactylation sensitizes GSCs to ES‐Cu‐induced cuproptosis. (A, B) DLAT oligomer formation in MES505 and MES21 cells expressing DLD WT or K445R following ES‐Cu treatment. (C–F) Representative immunofluorescence images and quantification of mitochondrial DLAT foci in MES505 and MES21 cells expressing DLD WT or K445R. Scale bars, 10 µm. (G) ES‐Cu dose‐response curves of MES505 cells expressing DLD WT or K445R. (H, I) Neurosphere formation and limiting dilution analyses of DLD WT‐ and K445R‐expressing MES505 cells in the presence or absence of ES‐Cu. (J) Expression of mesenchymal stemness‐associated markers in MES505 cells expressing HA‐DLD WT or K445R with or without ES‐Cu treatment. (K, L) Representative bioluminescence images and quantification of tumor burden in MES505‐derived orthotopic xenografts expressing DLD WT or K445R. (M) Representative immunohistochemical staining of DLD‐K445la, DLAT, and lipoic acid in tumor sections from the indicated groups. Scale bars, 200 µm. (N) Kaplan–Meier survival analysis of mice bearing DLD WT‐ or K445R‐expressing orthotopic tumors treated with or without ES‐Cu. Data are presented as mean ± SD. n = 3 biologically independent samples for D, F, and G; n = 5 independent sphere cultures for H; n = 8 biologically independent animals for L. P values were determined by two‐way ANOVA with Tukey's multiple‐comparison test for D, F, H, and L, nonlinear regression with extra sum‐of‐squares F test for G, ELDA chi‐square test for I, and log‐rank (Mantel–Cox) test for N. Original unprocessed blot images are provided in Figure S12C.

We next investigated the functional consequences of disrupting DLD lactylation. Expression of DLD K445R significantly increased ES‐Cu sensitivity in MES505 cells, as reflected by a pronounced leftward shift in the dose‐response curve (Figure 4G). Similar results were observed in MES21 cells (Figure S7G). K445R expression also markedly enhanced ES‐Cu‐mediated suppression of neurosphere formation and reduced stem‐cell frequency as determined by limiting dilution analysis (Figure 4H,I and Figure S7H,I). Accompanying this reduction in stem‐like traits, ES‐Cu treatment induced a more profound decrease in CD44 and ALDH1A3 expression and attenuated STAT3 signaling, as indicated by reduced phosphorylated STAT3 without obvious changes in total STAT3, in K445R‐expressing cells compared with WT controls (Figure 4J and Figure S7J). These data indicate that blockade of DLD K445 lactylation not only enhances cuproptotic responses but also compromises the maintenance of mesenchymal‐like GSC identity under copper stress. To determine whether these effects extend in vivo, we established orthotopic xenograft models using MES505‐derived GSCs expressing WT or K445R DLD. K445R expression alone modestly reduced intracranial tumor growth, whereas the combination of K445R expression and ES‐Cu treatment produced a substantially greater antitumor effect than either intervention alone, as assessed by longitudinal bioluminescence imaging (Figure 4K,L). Histopathological analyses further revealed reduced DLD‐K445 lactylation accompanied by increased DLAT and lipoic acid staining in K445R‐expressing tumors, particularly following ES‐Cu treatment (Figure 4M), providing in vivo evidence that disruption of DLD lactylation restores the lipoylated substrate pool required for cuproptosis. In agreement with these results, mice bearing K445R‐expressing tumors exhibited prolonged survival compared with mice bearing WT tumors, and the survival benefit was greatest when K445R expression was combined with ES‐Cu therapy (Figure 4N). Similar therapeutic effects were observed in MES21‐derived orthotopic models (Figure S7K–N).

The data establish DLD K445 lactylation as a previously unrecognized determinant of cuproptosis resistance in mesenchymal‐like GSCs. Genetic blockade of this modification restores DLAT aggregation, enhances copper‐induced cytotoxicity, suppresses stem‐like tumor programs, and markedly improves therapeutic responses to ES‐Cu in vivo.

### 2.7 SIRT5 is the Delactylase for DLD K445

The Sirtuin family has been increasingly recognized as the major delactylase based on accumulating evidence [24, 25, 26]. To investigate whether DLD K445 lactylation is dynamically regulated, we first examined the effect of nicotinamide (NAM), a broad inhibitor of sirtuin family deacylases [27, 28]. NAM treatment markedly increased DLD K445 lactylation in MES505 cells, suggesting that a member of the sirtuin family may function as a DLD delactylase (Figure 5A). In contrast, treatment with nicotinamide mononucleotide (NMN), a precursor of NAD^+^ that enhances sirtuin activity [29], significantly reduced DLD K445 lactylation (Figure 5B). Similar results were observed in MES21 cells (Figure S8A,B). This pattern suggested the existence of an NAD^+^‐dependent delactylation mechanism that counterbalances AARS2‐mediated DLD lactylation.

**FIGURE 5:**
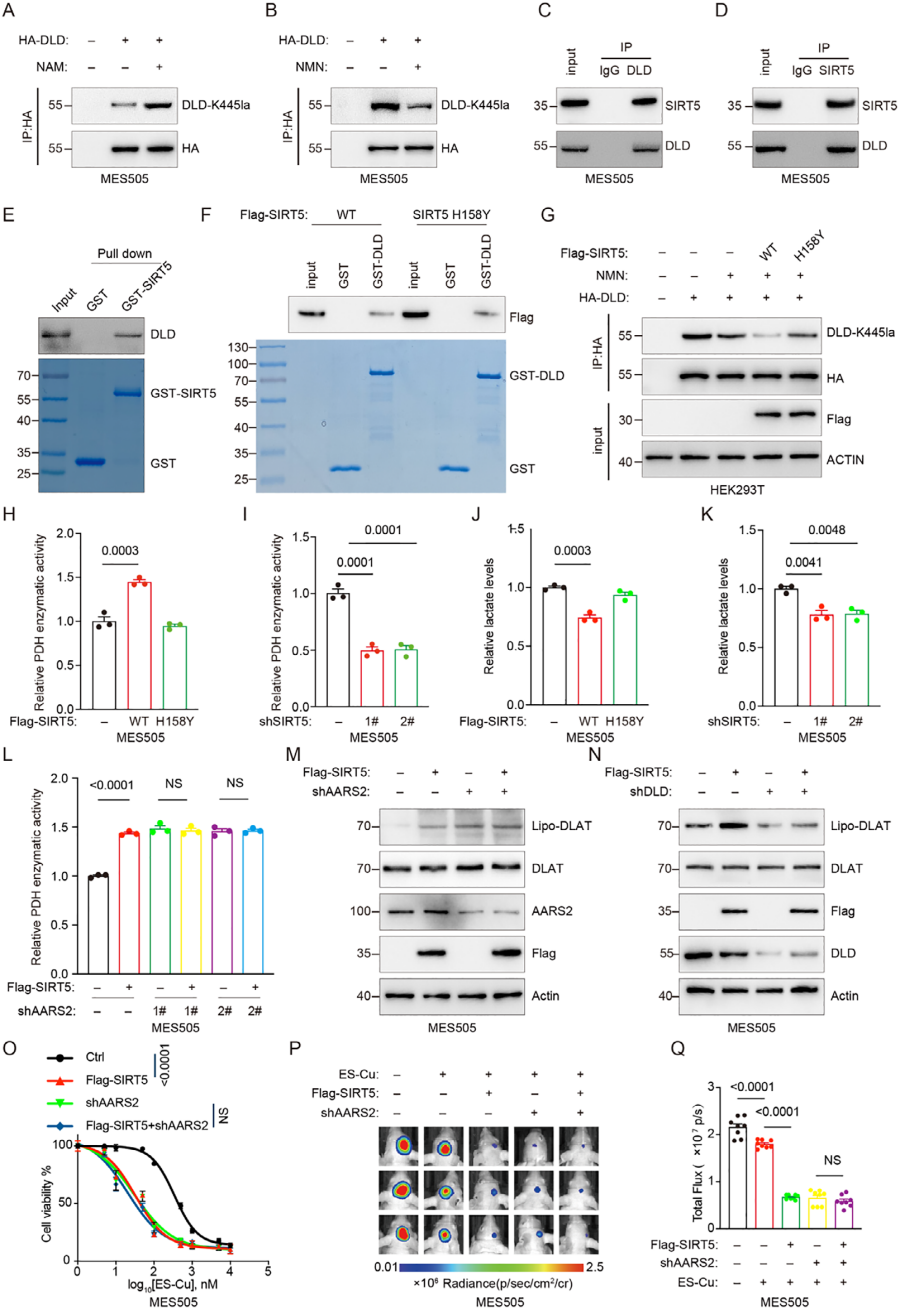
SIRT5 counteracts DLD K445 lactylation and restores cuproptosis sensitivity. (A, B) DLD K445 lactylation in HA‐DLD immunoprecipitates from MES505 cells treated with nicotinamide (NAM) or nicotinamide mononucleotide (NMN). (C, D) Reciprocal co‐immunoprecipitation analyses of endogenous DLD and SIRT5 in MES505 cells. (E, F) GST pull‐down assays demonstrating direct binding between DLD and WT or catalytically inactive SIRT5 H158Y. (G) DLD K445 lactylation in HEK293T cells expressing WT or H158Y SIRT5 in the presence or absence of NMN. (H, I) Relative PDH activity following expression of WT or H158Y SIRT5, or SIRT5 knockdown, in MES505 cells. (J, K) Intracellular lactate levels following expression of WT or H158Y SIRT5, or SIRT5 knockdown, in MES505 cells. (L) Relative PDH activity in MES505 cells expressing Flag‐SIRT5 with or without AARS2 knockdown. (M, N) DLAT lipoylation in MES505 cells expressing Flag‐SIRT5 in combination with AARS2 or DLD depletion. (O) ES‐Cu dose‐response curves of MES505 cells expressing Flag‐SIRT5 and/or shAARS2. (P, Q) Representative bioluminescence images and quantification of tumor burden in MES505‐derived orthotopic xenografts from the indicated treatment groups. Data are presented as mean ± SD. n = 3 biologically independent samples for H–L and O; n is indicated by individual data points for Q. P values were determined by one‐way ANOVA with Tukey's multiple‐comparison test for H–L and Q, and by nonlinear regression with an extra sum‐of‐squares F test for O. Original unprocessed blot images are provided in Figure S12D.

To identify the responsible enzyme, we interrogated DLD‐interacting proteins detected by mass spectrometry. Peptides derived from SIRT5 were identified among DLD‐associated proteins (Figure S8C and Table S2). Reciprocal co‐immunoprecipitation confirmed endogenous interaction between DLD and SIRT5 in MES505 and MES21 cells (Figure 5C,D and Figure S8D,E). GST pull‐down assays further demonstrated direct binding between DLD and SIRT5 in vitro, and the catalytically inactive SIRT5 H158Y mutant retained DLD‐binding capacity comparable to WT SIRT5 (Figure 5E,F), indicating that substrate recognition occurs independently of catalytic activity.

We therefore tested whether SIRT5 enzymatically regulates DLD lactylation. Expression of WT SIRT5 markedly reduced DLD K445 lactylation, whereas the catalytically inactive H158Y mutant failed to efficiently remove this modification, even under NMN stimulation (Figure 5G). This result identifies SIRT5 as a functional DLD K445 delactylase. In parallel with DLD delactylation, WT SIRT5 significantly increased PDH activity, whereas H158Y exhibited minimal effects. Conversely, SIRT5 depletion suppressed PDH activity in both MES505 and MES21 cells (Figure 5H,I and Figure S8F,G). SIRT5 manipulation also altered intracellular lactate levels, suggesting that DLD delactylation not only regulates PDH activity but also feeds back to influence cellular metabolic state (Figure 5J,K and Figure S8H,I).

Mechanistically, the effects of SIRT5 converged on the AARS2–DLD axis. SIRT5‐induced activation of PDH was maintained after AARS2 knockdown but was not further enhanced, indicating that AARS2 loss and SIRT5 activation act through a common DLD lactylation‐dependent mechanism (Figure 5L and Figure S8J). A similar pattern was observed for DLAT lipoylation, whereas DLD depletion largely abolished this effect (Figure 5M,N and Figure S8K,L), positioning DLD as the essential downstream effector through which SIRT5 regulates PDH complex function.

Functionally, SIRT5 overexpression significantly increased sensitivity to ES‐Cu, while combined SIRT5 expression and AARS2 knockdown failed to produce additive effects, consistent with pathway convergence (Figure 5O and Figure S8M). In orthotopic xenograft models, SIRT5 overexpression or AARS2 depletion each enhanced the therapeutic efficacy of ES‐Cu, reduced intracranial tumor burden, and prolonged survival. Importantly, simultaneous SIRT5 activation and AARS2 knockdown did not confer additional benefit, further supporting their operation within a shared signaling axis (Figure 5P,Q and Figure S8N–Q).

The data define SIRT5 as the NAD^+^‐dependent eraser of DLD K445 lactylation and reveal a reversible AARS2–DLD–SIRT5 lactylation circuit that controls PDH activity, DLAT lipoylation, and cuproptosis sensitivity in mesenchymal‐like GSCs.

### 2.8 Metabolic Modulation of DLD Lactylation Enhances ES‐Cu Efficacy in GSC‐Derived Models

Because DLD K445 lactylation is governed by both lactate availability and NAD^+^‐dependent delactylation, we next asked whether pharmacological manipulation of these metabolic pathways could be leveraged to enhance cuproptosis‐based therapy. In MES505 and MES21 cells, NMN markedly increased sensitivity to ES‐Cu, whereas NALA shifted the dose‐response curves toward resistance. NMN largely reversed the protective effect conferred by NALA, indicating that activation of NAD^+^‐dependent delactylation can counteract lactate‐driven cuproptosis resistance (Figure 6A,B).

**FIGURE 6:**
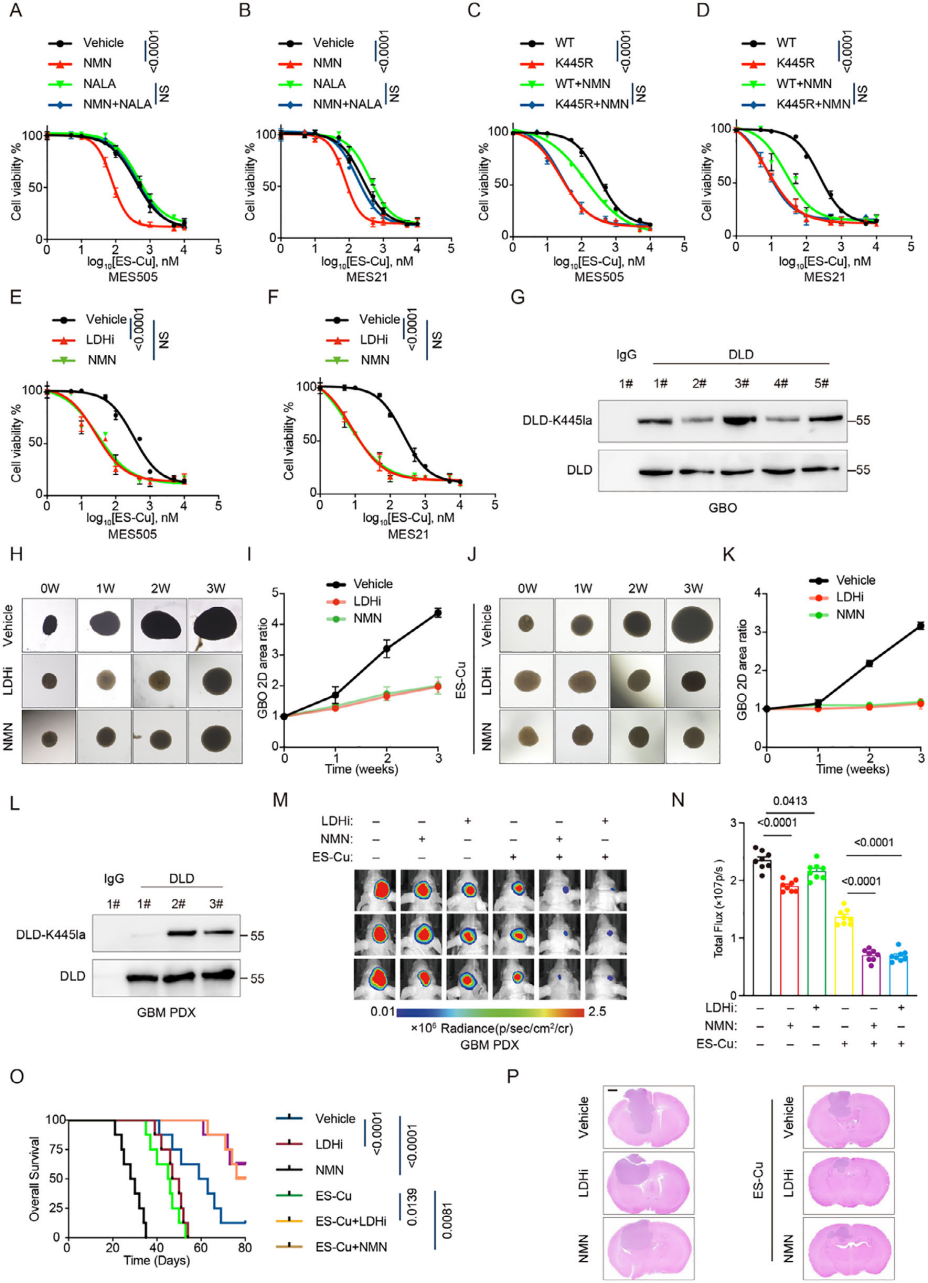
Metabolic targeting of DLD K445 lactylation enhances ES‐Cu sensitivity in GSC‐derived models. (A, B) ES‐Cu dose‐response curves of MES505 and MES21 cells treated with vehicle, NMN, NALA, or the combination of NMN and NALA. (C, D) ES‐Cu dose‐response curves of MES505 and MES21 cells expressing DLD WT or K445R in the presence or absence of NMN. (E, F) ES‐Cu dose‐response curves of MES505 and MES21 cells treated with vehicle, LDH inhibitor (LDHi), or NMN. (G) Endogenous DLD K445 lactylation detected in patient‐derived glioblastoma organoids (GBOs) by DLD immunoprecipitation. (H, I) Representative GBO images and quantification of organoid growth following treatment with vehicle, LDHi, or NMN. (J, K) Representative GBO images and quantification of organoid growth following ES‐Cu treatment in combination with vehicle, LDHi, or NMN. (L) Endogenous DLD K445 lactylation detected in GBM patient‐derived xenograft (PDX) specimens by DLD immunoprecipitation. (M, N) Representative bioluminescence images and quantification of tumor burden in MES505‐derived orthotopic xenografts treated with LDHi or NMN, with or without ES‐Cu. (O) Kaplan–Meier survival analysis of mice bearing MES505‐derived orthotopic xenografts treated as indicated. (P) Representative hematoxylin and eosin (H&E)‐stained brain sections from mice treated with vehicle, LDHi, or NMN, in the presence or absence of ES‐Cu. Scale bars, 2 mm. Data are presented as mean ± SD. n = 3 biologically independent samples for A–F, I, and K; n is indicated by individual data points for N. P values were determined by nonlinear regression with extra sum‐of‐squares F test for A–F, two‐way repeated‐measures ANOVA with Tukey's multiple‐comparison test for I and K, one‐way ANOVA with Tukey's multiple‐comparison test for N, and log‐rank (Mantel–Cox) test for O. Original unprocessed blot images are provided in Figure S12E.

To determine whether this effect depends on DLD K445 lactylation, we compared DLD WT‐ and K445R‐expressing cells. NMN significantly sensitized WT cells to ES‐Cu but failed to further enhance sensitivity in K445R‐expressing cells, indicating that the therapeutic effect of NMN is mediated predominantly through reversal of DLD K445 lactylation (Figure 6C,D). Both LDH inhibition and NMN increased ES‐Cu responsiveness in MES505 and MES21 cells, supporting the notion that metabolic interventions targeting either lactate production or lactylation turnover converge on a common DLD‐dependent mechanism (Figure 6E,F).

We next extended these observations to patient‐derived glioblastoma organoids (GBOs). Immunoprecipitation analyses detected endogenous DLD K445 lactylation across independent GBO specimens, although the abundance of this modification varied substantially among tumors (Figure 6G). Among these samples, GBO #3 exhibited the highest level of DLD K445 lactylation and was therefore selected for subsequent functional studies. In this DLD‐K445la‐high organoid model, both LDH inhibition and NMN modestly suppressed organoid expansion under basal conditions, indicating that DLD lactylation contributes to maintenance of GBO growth (Figure 6H,I). Beyond their basal effects on organoid growth, both interventions markedly potentiated the growth‐inhibitory effects of ES‐Cu, resulting in sustained suppression of organoid progression over time (Figure 6J,K). These results demonstrate that metabolic targeting of DLD lactylation enhances cuproptosis induction in a patient‐derived model that preserves key features of tumor architecture and cellular heterogeneity.

We further evaluated the translational potential of this strategy in patient‐derived xenograft models. Endogenous DLD K445 lactylation was readily detected in GBM PDX tumors (Figure 6L), confirming that this modification is maintained in patient‐derived in vivo models. In orthotopic intracranial models, LDH inhibition or NMN reduced tumor burden, whereas combination with ES‐Cu produced substantially greater suppression of bioluminescent tumor growth than either intervention alone (Figure 6M,N). In line with these results, both LDH inhibition and NMN significantly prolonged survival in ES‐Cu‐treated mice (Figure 6O). Histological examination of brain sections further supported the antitumor efficacy of these regimens without overt pathological abnormalities in the examined tissues (Figure 6P).

These experiments show that pharmacological reduction of DLD K445 lactylation sensitizes GSC‐derived organoid and intracranial tumors to cuproptosis induction. The data establish DLD lactylation as a therapeutically actionable metabolic checkpoint and provide a rationale for combining metabolic intervention with copper ionophore‐based strategies for glioblastoma treatment.

### 2.9 K445‐Targeting Peptides Suppress DLD Lactylation and Overcome Cuproptosis Resistance

Because pharmacological modulation of lactate or NAD^+^ metabolism may influence multiple cellular pathways beyond DLD lactylation, we next sought to develop a more selective strategy to directly target the DLD K445 lactylation site. Guided by the amino acid sequence surrounding K445, we designed and synthesized five short cell‐penetrating peptides predicted to interfere with modification of this residue (Figure S10A). Among these candidates, peptide 5 exhibited the strongest inhibitory effect on DLD K445 lactylation and was therefore selected for subsequent studies as K445‐pe (Figure S10B). To control for sequence‐specific effects, we generated a matched mutant peptide in which the K445‐corresponding lysine residue was replaced by arginine (K445R‐pe). Biotinylated peptide pull‐down assays demonstrated that K445‐pe specifically interacted with endogenous DLD, whereas the K445R‐pe mutant showed markedly reduced binding (Figure S9A). This interaction was further confirmed by competition assays using excess unlabeled peptide and by direct binding assays with purified DLD protein (Figure S9B,C). Moreover, K445‐pe disrupted the endogenous interaction between AARS2 and DLD, suggesting that K445‐pe binding to DLD interferes with AARS2–DLD association and thereby suppresses AARS2‐mediated DLD lactylation. (Figure S9D). K445‐pe suppressed DLD K445 lactylation in a dose‐dependent manner, whereas K445R‐pe showed minimal inhibitory activity (Figure S10C,D). Consistent with on‐target activity, K445‐pe attenuated both basal and ES‐Cu‐induced DLD K445 lactylation in HA‐DLD‐expressing cells, while DLD K445R expression confirmed the site specificity of this effect (Figure 7A,B). K445‐pe also increased DLAT lipoylation in MES505 and MES21 cells, whereas K445R‐pe failed to induce comparable changes (Figure 7C and Figure S10E).

**FIGURE 7:**
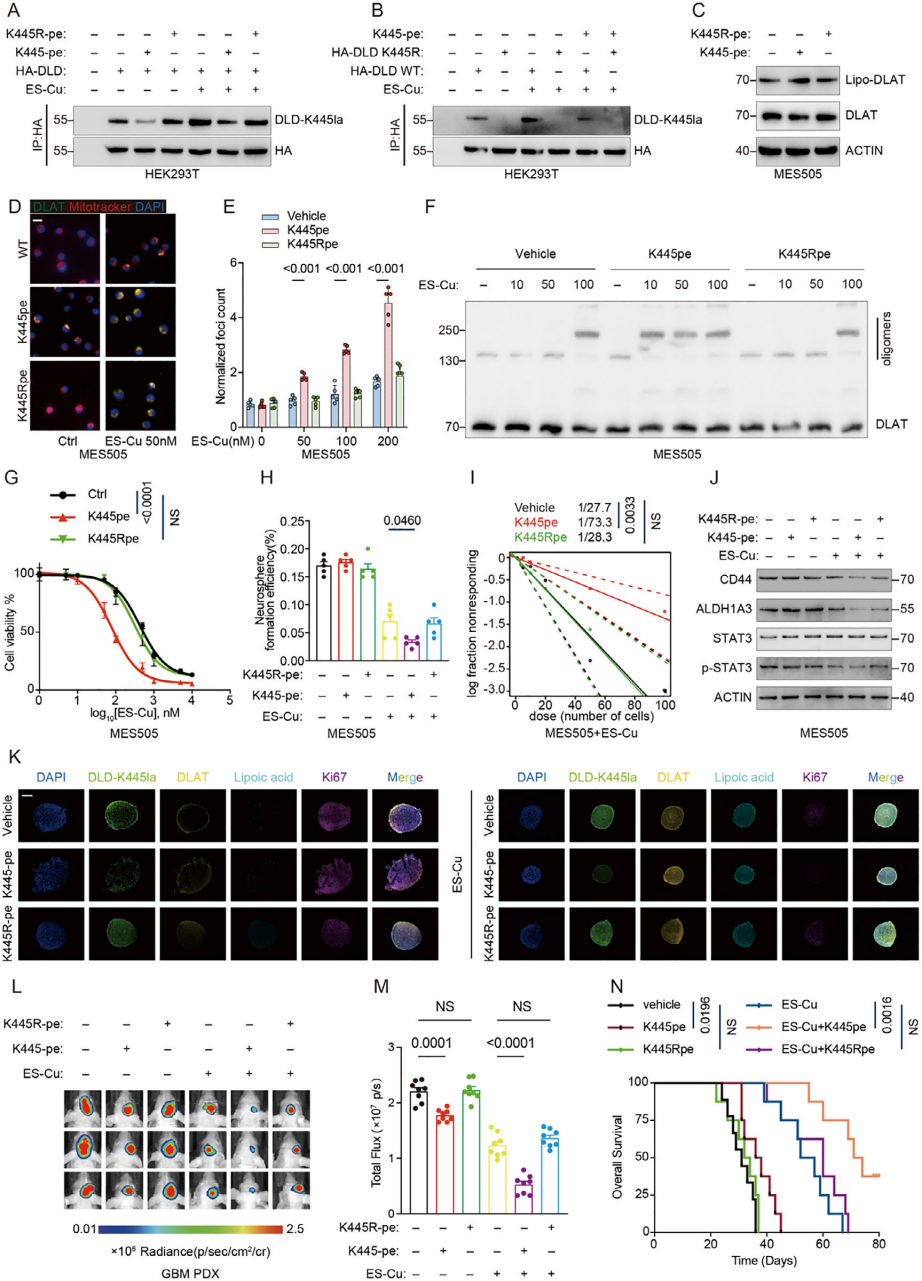
Direct targeting of DLD K445 lactylation restores cuproptosis sensitivity in glioblastoma stem‐like cells. (A) DLD K445 lactylation in HA‐DLD immunoprecipitates from HEK293T cells exposed to K445‐pe, K445R‐pe, and/or ES‐Cu. (B) DLD K445 lactylation in HEK293T cells expressing HA‐DLD WT or K445R in the presence or absence of K445‐pe and ES‐Cu. (C) DLAT lipoylation in MES505 cells following treatment with K445‐pe or K445R‐pe. (D, E) Representative immunofluorescence images and quantification of mitochondrial DLAT foci in MES505 cells exposed to vehicle, K445‐pe, or K445R‐pe following ES‐Cu treatment. Scale bars, 10 µm. (F) DLAT oligomer formation in MES505 cells treated with increasing concentrations of ES‐Cu in the presence of vehicle, K445‐pe, or K445R‐pe. (G) ES‐Cu dose‐response curves of MES505 cells treated with vehicle, K445‐pe, or K445R‐pe. (H, I) Neurosphere formation and limiting dilution analyses of MES505 cells treated with K445‐pe or K445R‐pe in the presence or absence of ES‐Cu. (J) Expression of mesenchymal stemness‐associated markers in MES505 cells under the indicated treatment conditions. (K) Multiplex immunofluorescence staining of DLD‐K445la, DLAT, lipoic acid, and Ki67 in patient‐derived glioblastoma organoids following treatment with K445‐pe or K445R‐pe, with or without ES‐Cu. Scale bars, 100 nm. (L, M) Representative bioluminescence images and quantification of tumor burden in GBM patient‐derived xenograft (PDX) models receiving the indicated treatments. (N) Kaplan–Meier survival analysis of mice bearing GBM PDX tumors treated as indicated. Data are presented as mean ± SD. n = 3 biologically independent samples for E, G, and H; n is indicated by individual data points for M. P values were determined by two‐way ANOVA with Tukey's multiple‐comparison test for E, H, and M, nonlinear regression with extra sum‐of‐squares F test for G, ELDA chi‐square test for I, and log‐rank (Mantel–Cox) test for N. Original unprocessed blot images are provided in Figure S12F.

We next examined whether direct blockade of DLD K445 lactylation could restore cuproptosis susceptibility. K445‐pe markedly enhanced ES‐Cu‐induced DLAT foci formation and high‐molecular‐weight DLAT oligomerization, whereas K445R‐pe had little effect (Figure 7D–F and Figure S10F–H). Consistent with enhanced cuproptotic stress, K445‐pe shifted the ES‐Cu dose‐response curve leftward and significantly increased ES‐Cu sensitivity in both MES505 and MES21 cells (Figure 7G and Figure S10I). In MES505 cells, K445‐pe further potentiated ES‐Cu‐mediated suppression of neurosphere formation and reduced stem‐cell frequency, while K445R‐pe did not significantly alter these phenotypes (Figure 7H,I). Concordantly, combined K445‐pe and ES‐Cu treatment suppressed stemness‐associated markers (CD44 and ALDH1A3) and reduced STAT3 activation, as evidenced by decreased phosphorylated STAT3 levels (Figure 7J). Comparable results were obtained in MES21 cells, supporting the reproducibility of this effect across independent mesenchymal‐like GSC models (Figure S10J–L).

We then evaluated the therapeutic potential of K445‐pe in patient‐derived glioblastoma organoid and xenograft models. In GBOs, K445‐pe alone moderately restrained organoid growth, whereas combination treatment with ES‐Cu produced substantially greater suppression of organoid expansion than either intervention alone (Figure S10M–P). Multiplex immunofluorescence analyses further demonstrated reduced DLD‐K445la and Ki67 staining accompanied by increased DLAT and lipoic acid signals following K445‐pe treatment, particularly in the presence of ES‐Cu (Figure 7K and Figure S11A–D), indicating effective target engagement and restoration of the cuproptosis‐associated molecular program. In orthotopic GBM PDX models, K445‐pe significantly enhanced the antitumor efficacy of ES‐Cu, resulting in reduced tumor burden and prolonged survival compared with monotherapy‐treated controls (Figure 7L–N). Response‐surface modeling and synergy analyses further demonstrated cooperative interactions between K445‐pe and ES‐Cu in both MES505 and MES21 cells (Figure S11E–H). Histopathological examination together with serum biochemical analyses revealed no evidence of overt systemic toxicity associated with K445‐pe treatment alone or in combination with ES‐Cu (Figure S11I–R).

These results nominate DLD K445 lactylation as a therapeutically actionable vulnerability in mesenchymal‐like glioblastoma stem cells and provide proof‐of‐concept evidence that direct pharmacological disruption of this modification can restore cuproptosis sensitivity and enhance the efficacy of copper‐dependent therapeutic strategies.

### 2.10 DLD K445 Lactylation Correlates With Poor Clinical Outcomes in Glioblastoma

To investigate the clinical relevance of DLD K445 lactylation in glioblastoma, we analyzed DLD K445 lactylation levels in a previously established glioblastoma tissue microarray cohort comprising 138 patient specimens. Immunohistochemical staining revealed heterogeneous expression of DLD K445 lactylation across glioblastoma samples, which were stratified into DLD K445la‐high and DLD K445la‐low groups based on staining intensity. Patients with high DLD K445 lactylation exhibited significantly shorter overall survival and progression‐free survival compared with those with low DLD K445 lactylation levels (Figure 8A–C). These findings indicate that elevated DLD K445 lactylation is associated with aggressive clinical behavior and poor prognosis in glioblastoma.

**FIGURE 8:**
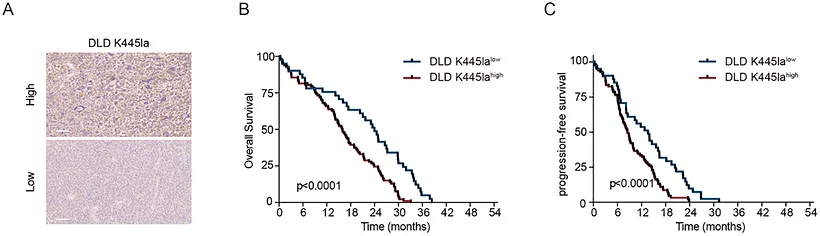
Clinical relevance of DLD K445 lactylation in glioblastoma patients. (A) Representative immunohistochemical staining images showing high and low levels of DLD K445 lactylation (DLD K445la) in a glioblastoma tissue microarray cohort. Scale bars: 100 µm. (B, C) Kaplan–Meier survival analyses showing the association between DLD K445la levels and overall survival (B) and progression‐free survival (C) of glioblastoma patients. Patients were stratified into DLD K445la^high and DLD K445la^low groups according to the predefined cutoff value. Statistical significance was determined using the log‐rank test.

Collectively, our findings establish a lactate‐responsive DLD K445 lactylation axis that regulates cuproptosis sensitivity in glioblastoma. Under metabolic conditions characterized by enhanced glycolytic flux and lactate accumulation, AARS2‐mediated DLD K445 lactylation suppresses PDH enzymatic activity and decreases DLAT lipoylation, thereby limiting cuproptosis‐associated DLAT aggregation and promoting cuproptosis resistance. Conversely, inhibition of DLD K445 lactylation through SIRT5‐mediated delactylation or the K445‐pe targeting peptide restores PDH activity, enhances DLAT lipoylation, and sensitizes glioblastoma cells to cuproptosis induction.

## 3 Discussion

We demonstrate DLD K445 lactylation as a metabolic safeguard against ES‐Cu‐triggered cuproptosis in mesenchymal GSCs (Figure 8). Combining single‐cell RNA‐seq, quantitative lactylome, and functional assays, lactate signaling was enriched in cuproptosis‐resistant stem tumor cells, most prominently in the mesenchymal subtype. Mechanistically, AARS2 catalyzes DLD K445 lactylation to inhibit PDH activity and DLAT lipoylation; SIRT5 erases this mark to rescue cuproptosis sensitivity. This reversible axis connects lactate metabolism, mitochondrial lipoylation, and copper‐mediated cell death via site‐specific modification of a mitochondrial metabolic enzyme.

Copper is an indispensable trace element, but excess intracellular copper drives cuproptosis via aggregation of lipoylated mitochondrial proteins. We detected higher copper contents in GBM tissues, correlating with worse patient survival, confirming a copper‐rich niche and supporting cuproptosis‐targeted therapy. Nonetheless, tumor cell metabolic status, rather than copper concentration alone, dominates cuproptosis susceptibility.

Our work highlights lactate‐driven metabolic adaptation as a key driver of GSC cuproptosis resistance. Cuproptosis relies on mitochondrial lipoylation and copper‐triggered aggregation of lipoylated proteins. Mesenchymal GSCs had higher LactateScore and CuproResScore, and lactate metabolic disruption drastically altered ES‐Cu response. Lactate metabolic rewiring is not just a marker of malignant GBM, but a functional pathway suppressing cuproptosis, explaining the low sensitivity of glycolysis‐high glioma cells to copper ionophores.

Recent studies have highlighted the importance of post‐translational modifications (PTMs) in regulating cuproptosis. For example, AKT1‐mediated phosphorylation of FDX1 promotes resistance to cuproptosis in triple‐negative breast cancer [6]. Lactylation of METTL16 promotes cuproptosis via m6A modification on FDX1 mRNA in gastric cancer [7]. NUDT21 lactylation reprograms alternative polyadenylation to promote cuproptosis resistance. In addition, carnitine palmitoyltransferase 1A (CPT1A) induces succinylation of DLD at lysine 320, thereby promoting cuproptosis in hepatic stellate cells [30]. Collectively, these studies support an emerging model in which cuproptosis is dynamically regulated by PTM‐dependent metabolic signaling. Our findings extend this framework by identifying DLD K445 lactylation as a previously unrecognized mitochondrial modification that directly links lactate metabolism to cuproptosis resistance in glioblastoma stem cells.

Our data reveal DLD K445 lactylation modulates PDH catalytic activity without disrupting PDH complex assembly or DLD protein stability. K445R mutant still binds PDH subunits, and molecular dynamics ruled out severe complex destabilization. Loss of K445 lactylation elevates PDH activity, DLAT lipoylation, and mitochondrial oxidative metabolism. DLD K445 lactylation fine‐tunes PDH catalytic capacity to control lipoylated DLAT levels, governing copper‐triggered DLAT aggregation and cuproptosis.

AARS2 and SIRT5 oppositely control DLD K445 lactylation, broadening the known roles of lactylation in tumor metabolism. The reversible AARS2–DLD–SIRT5 cascade links lactate levels and NAD^+^‐dependent delactylation to cuproptosis susceptibility, proving mitochondrial lactylation acts as an active signal rather than a waste lactate metabolite.

Therapeutically, DLD K445 lactylation is a targetable driver of cuproptosis resistance. Suppressing lactate synthesis or boosting NAD^+^‐mediated delactylation sensitizes cells to ES‐Cu yet lacks specificity. We generated the cell‐penetrating peptide K445‐pe to selectively block the DLD K445 lactylation motif. K445‐pe downregulates DLD lactylation, recovers DLAT lipoylation, facilitates copper‐mediated DLAT aggregation, and restores cuproptosis sensitivity in GSCs in vitro and in vivo. It synergizes with ES‐Cu to suppress tumor growth in patient‐derived organoids and intracranial xenografts, showing great translational value. Thus, DLD K445 lactylation is both a core metabolic adaptation switch and a druggable vulnerability in GBM.

This work has several limitations. Other enzymes or metabolic microenvironments may regulate DLD K445 lactylation in different GBM cell states. The global lactylome includes additional potential cuproptosis‐related substrates that may cooperate with DLD to control copper sensitivity. Despite the robust anti‐tumor effects of K445‐pe in preclinical models, further optimization of peptide stability, pharmacokinetics, blood‐brain barrier permeability, and delivery strategies is required for future clinical application. Previous studies have demonstrated that cell‐penetrating peptides and peptide‐based delivery platforms can facilitate BBB transport and intracranial therapeutic delivery [31]. Although BBB permeability of K445‐pe was not directly quantified in this study, the therapeutic efficacy observed in orthotopic GBM models suggests that K445‐pe reaches the intracranial tumor microenvironment and retains on‐target pharmacological activity. GBM intertumoral heterogeneity may create variable reliance on the AARS2–DLD–SIRT5 axis; therefore, DLD K445 lactylation and DLAT lipoylation could serve as potential biomarkers for patient subgrouping and treatment guidance.

In conclusion, lactate metabolism confers cuproptosis resistance to GSCs via AARS2‐catalyzed DLD K445 lactylation. This modification inhibits PDH activity and DLAT lipoylation, reducing copper‐induced DLAT aggregation and cuproptosis. Genetic, metabolic, and peptide‐mediated inhibition of DLD K445 lactylation rescues cuproptosis responsiveness and amplifies ES‐Cu anti‐tumor effects in multiple GBM models. Peptides masking the DLD K445 lactylation motif effectively reverse cuproptosis resistance, supporting lactylation‐targeted therapy as a promising glioblastoma treatment strategy.

## 4 Methods and Materials

### 4.1 Human Glioblastoma Specimens

Human glioblastoma specimens and non‐neoplastic brain tissues were obtained from patients who underwent surgical resection at the First Affiliated Hospital of Nanjing Medical University. Tumors were diagnosed independently by two neuropathologists according to the 2021 World Health Organization classification of central nervous system tumors. Written informed consent was obtained from all patients before sample collection. The study was approved by the Institutional Ethics Committee of the First Affiliated Hospital of Nanjing Medical University and conducted in accordance with the Declaration of Helsinki. Fresh tumor specimens were used for the generation of glioblastoma organoids (GBOs), patient‐derived glioblastoma stem cell cultures, and molecular analyses. Formalin‐fixed paraffin‐embedded specimens were used for immunohistochemical and immunofluorescence studies. In addition, a tissue microarray consisting of 138 formalin‐fixed, paraffin‐embedded GBM tissues was obtained from the Department of Neurosurgery, Second and Fourth Affiliated Hospitals of Harbin Medical University for clinical correlation analyses [32, 33], The use of these archived human specimens was approved by the Institutional Ethics Committee of Harbin Medical University, and all samples were obtained with written informed consent from patients or in accordance with institutional policies for retrospective tissue studies.

### 4.2 Cell Culture

Human glioma cell lines U87 and T98 were cultured in high‐glucose DMEM medium supplemented with 10% fetal bovine serum (FBS) and penicillin‐streptomycin antibiotics. All glioblastoma stem‐like cell lines (PN35, PN182, AC14, AC71, OPC27, OPC58, MES21, MES505) were maintained as floating neurospheres in serum‐free DMEM/F12 neural stem cell medium containing B27 supplement, recombinant human epidermal growth factor (EGF) and basic fibroblast growth factor (bFGF), plus penicillin‐streptomycin. The HEK293T cell line was propagated in high‐glucose DMEM supplemented with 10% FBS and matching antibiotics. All cells were incubated at 37°C in a humidified incubator with 5% CO2 atmosphere, and routine mycoplasma testing was performed to ensure all cell lines were free of mycoplasma contamination before IC50 pharmacological assays.

### 4.3 Animals

Female BALB/c nude mice (4–6 weeks old) were purchased from GemPharmatech Co., Ltd. (Nanjing, China). All animal procedures were approved by the Institutional Animal Care and Use Committee of Nanjing Medical University and performed in accordance with institutional guidelines and NIH recommendations for the care and use of laboratory animals. Mice were randomly assigned to experimental groups (n = 8 per group).

For intracranial xenograft studies, patient‐derived glioblastoma stem cells (GSCs) or patient‐derived xenograft (PDX) cells expressing firefly luciferase were dissociated into single‐cell suspensions and stereotactically implanted into the right cerebral hemisphere of nude mice (1 × 10^5^ cells per mouse in 5 µL PBS) at a depth of approximately 2.0 mm. Tumor growth was monitored weekly using an in vivo imaging system (IVIS, PerkinElmer). Bioluminescence signals were quantified according to the manufacturer's instructions. Mice were monitored daily and euthanized upon the development of neurological symptoms or when predefined humane endpoints were reached. Brain tissues were collected for histological and immunohistochemical analyses.

### 4.4 Antibodies and Reagents

The following antibodies and reagents were used in this study: anti‐β‐actin (AF0003, Beyotime), anti‐LDHA (19987‐1‐AP, Proteintech), anti‐L‐lactyllysine (PTM‐1401RM, PTM Bio), anti‐AARS2 (22696‐1‐AP, Proteintech), anti‐DLAT (ab126224, Abcam; 12362S, Cell Signaling Technology), anti‐DLD (16431‐1‐AP, Proteintech), anti‐DLD‐K445la (N/A, Arolab), anti‐lipoic acid (ab58724, Abcam), anti‐DLST (5556S, Cell Signaling Technology), anti‐Ki‐67 (ab15580, Abcam), anti‐Flag (TT0053, Abmart), and anti‐HA (ab9110, Abcam). Chemical reagents included the copper ionophore elesclomol (HY‐12040, MedChemExpress), sodium L‐lactate (71718, Sigma–Aldrich), L‐lactic acid (L1750, Sigma–Aldrich), sodium oxamate (O2751, Sigma–Aldrich), syrosingopine (S9907, Selleckchem), a GLO1 inhibitor (HY‐15167A, MedChemExpress), and nicotinamide mononucleotide (NMN; N3501, Sigma–Aldrich).

### 4.5 Mass Spectrometry Analysis of DLD‐Interacting Proteins

HEK293T cells transfected with HA‐DLD were lysed and subjected to immunoprecipitation using an anti‐DLD antibody or control IgG. The immune complexes were captured with Protein A/G agarose beads (P2197, Beyotime). After three washes with lysis buffer, the bound proteins were eluted by boiling in 1 × SDS loading buffer and separated by SDS–PAGE. Protein bands were excised from the gel and subjected to mass spectrometry analysis at the Beijing Genomics Institute (BGI, Beijing, China).

### 4.6 Copper Content Detection

Copper levels in glioma tissues were measured using a copper colorimetric assay kit (E‐BC‐K300‐M; Elabscience) according to the manufacturer's instructions. Briefly, tissue samples were homogenized in the assay buffer and centrifuged to obtain the supernatant. The copper concentration was determined by measuring absorbance at 580 nm using a microplate reader and calculated based on a standard curve generated with the provided copper standards.

### 4.7 Single‐Cell RNA‐Sequencing Analysis

Single‐cell RNA‐sequencing data from human glioblastoma samples were analyzed using Seurat (v5.0) [34]. After quality‐control filtering, gene‐expression matrices were normalized and scaled, followed by principal component analysis (PCA). Batch effects among samples were corrected using Harmony [35], and cells were clustered using a shared nearest‐neighbor graph. Uniform Manifold Approximation and Projection (UMAP) was used for dimensionality reduction and visualization [36]. Cell populations were annotated according to established lineage markers. Major cell types included microglia, macrophages, monocytes, T/NK cells, oligodendrocytes, fibroblasts, dendritic cells, endothelial cells, neutrophils, B cells, and malignant tumor cells. Cell‐type assignments were further supported by differential gene‐expression analysis and canonical marker‐gene expression patterns. Malignant cells were subsequently divided into stem‐like and non‐stem‐like populations. Stem‐like malignant cells were further classified into OPC‐like, NPC/PN‐like, AC‐like, and MES‐like states based on previously reported GBM state‐specific gene signatures [3]. Module scores for each cellular state were calculated using the AddModuleScore function in Seurat. To evaluate cuproptosis resistance and lactate‐associated metabolic activity, CuproResScore and LactateScore were calculated for individual cells using predefined gene signatures. Score distributions were visualized on UMAP embeddings and compared across cell populations and malignant‐cell states. For sample‐level analyses, scores were averaged within each sample to avoid bias introduced by unequal cell numbers. Correlations between CuproResScore and LactateScore were assessed using Spearman correlation analysis. Comparisons between two groups were performed using the Wilcoxon rank‐sum test, whereas comparisons among multiple groups were performed using the Kruskal–Wallis test.

### 4.8 CCK‐8 Assay

Cell viability was assessed using the Cell Counting Kit‐8 (CCK‐8; CK04, Dojindo). GBM cells were seeded into 96‐well plates and cultured overnight to allow cell attachment. Subsequently, 10 µL of CCK‐8 reagent was added to each well containing 100 µL of culture medium and incubated at 37°C for 2 h. Absorbance was measured at 450 nm using a microplate reader (Thermo Fisher Scientific). All experiments were performed in triplicate.

### 4.9 Dot blot Assay

Synthetic peptides corresponding to the DLD K445 sequence (ILGQKSTDRV) and its lactylated counterpart (ILGQKlaSTDRV) were synthesized and dissolved at a concentration of 2 mg/mL. Serial dilutions of each peptide (1, 4, 16, and 64 ng) were spotted onto a nitrocellulose membrane. After air‐drying, the membrane was blocked with 5% nonfat milk in TBST buffer and then incubated with the indicated primary antibody followed by HRP‐conjugated secondary antibodies for immunoblot detection.

### 4.10 Measurement of Lactate Levels

Intracellular lactate levels were measured using a lactate colorimetric/fluorometric assay kit (ab65331, Abcam) according to the manufacturer's instructions. Briefly, cells were collected and lysed to obtain the supernatant, which was then subjected to the assay. Lactate concentrations were determined by measuring absorbance or fluorescence using a microplate reader according to the kit protocol.

### 4.11 Measurement of Mitochondrial Respiration and Glycolysis

Mitochondrial respiration and glycolytic activity were assessed by measuring oxygen consumption rate (OCR) and extracellular acidification rate (ECAR) using a Seahorse XFe96 Extracellular Flux Analyzer (Agilent) as previously described. Data were normalized to protein content determined by a bicinchoninic acid (BCA) assay or to cell confluency measured using the Incucyte Live‐Cell Analysis System (Sartorius).

### 4.12 Immunofluorescence

Cells were seeded onto glass coverslips placed in 24‐well plates and cultured overnight. Cells were then fixed, permeabilized, and blocked before incubation with the indicated primary antibodies at 4°C overnight. After washing with PBS, cells were incubated with appropriate fluorescent secondary antibodies for 1 h at room temperature and counterstained with DAPI to visualize nuclei. Fluorescence images were acquired using a Leica Stellaris STED microscope.

### 4.13 Foci Segmentation and Analysis

DLAT fluorescence underwent background subtraction via morphological opening. A Laplacian filter enhanced foci, followed by thresholding to generate binary masks for quantification of foci count, area, and intensity. Nuclei failing morphological and DAPI criteria were excluded to reduce segmentation artifacts; foci < 75 pixels were discarded as background. Foci counts were normalized to total cell number per well.

### 4.14 Molecular Dynamics Simulation

The crystal structure of mature truncated human DLD homodimer (PDB ID: 2F5Z) was retrieved from the RCSB PDB. Lactylation modification was computationally introduced at Lys‐410 (corresponding to full‐length Lys445) to generate the K445‐lactylated DLD model. All simulations were performed with GROMACS 2022 using the Amber99sb‐ildn protein force field and GAFF for modified residue topology. The system was solvated with SPCE water model and neutralized with counterions. Electrostatic interactions were processed via the PME algorithm, with van der Waals and Coulomb cutoffs set to 1.2 nm.

Steepest‐descent energy minimization (500,000 steps) was performed, followed by sequential 100 ps NVT and 100 ps NPT equilibration at 298.15 K and 1 bar. The production MD simulation ran for 100 ns under periodic boundary conditions with V‐rescale temperature coupling. Trajectory analysis, including RMSD, RMSF, Rg, hydrogen bond, and SASA calculations, was conducted via built‐in Gromacs modules. gmx_MMPBSA was applied to compute binding free energy and residue‐wise energy decomposition.

### 4.15 RNA Isolation and Quantitative Real‐Time PCR

Total RNA was extracted using TRIzol reagent (15596‐026, Invitrogen) according to the manufacturer's instructions. Complementary DNA (cDNA) was synthesized from 1 µg of total RNA using a reverse transcription kit (K1622, Thermo Fisher Scientific). Quantitative real‐time PCR (qRT‐PCR) was performed using SYBR Green Master Mix according to the manufacturer's protocol. Gene expression levels were normalized to β‐actin and calculated using the 2^−ΔΔCt^ method. The primer sequences used for qRT‐PCR were as follows:

**Table:** 

	Forward primer (5′‐3′)	Reverse primer (5′‐3′)
FDX1	CCACTTTATAAACCGTGATGGTG	ACATGCACCAAAGCCATCAA
LIPT1	CCTCTGTTGTAATTGGTAGGCAT	CTGGGGTTGGACAGCATTCAG
LIAS	CAGCCCAGTCAGACCGTTAAG	TTTCTGGCGTTTTAGGTTTCCT
DLD	CTGGCTCACAAAGCAGAGGAT	GCTGTTAGCAGCAAATGGGAA
DLAT	CCGCCGCTATTACAGTCTTCC	CTCTGCAATTAGGTCACCTTCAT
PDHA1	TGGTAGCATCCCGTAATTTTGC	ATTCGGCGTACAGTCTGCATC
PDHB	AAGAGGCGCTTTCACTGGAC	ACTAACCTTGTATGCCCCATCA
MTF1	GTGACTTGAGCCTTCTGTCCAC	TGAAATTGTGCTGAGGTCCTG
GLS	ACTTCTCAGGGCAGTTTGCTTTC	TATCCAGAGGAGGAGACCAGCAC
CDKN2A	CGACCCTGTCCCTCAAATCC	TATCGCGGAGGAAGGAAACG
ACTB	GTCCACCGCAAATGCTTCTA	TGCTGTCACCTTCACCGTTC

### 4.16 Generation of Patient‐Derived Glioblastoma Organoids (GBOs)

Glioblastoma organoids (GBOs) were generated from freshly resected patient tumor specimens as previously described [37]. Briefly, minced tumor tissues were cultured in ultra‐low attachment 6‐well plates with 4 mL GBO medium per well. GBO medium is a 1:1 mix of DMEM/F12 and Neurobasal, supplemented with 1 × GlutaMAX, non‐essential amino acids, penicillin–streptomycin, N2, vitamin A‐free B27, 1 × 2‐mercaptoethanol, and 2.5 µg/mL human insulin. Cultures were incubated at 37°C, 5% CO_2_, in a humidified atmosphere with shaking at 120 rpm; 75% of the medium was refreshed every 48 h. Spherical glioblastoma organoids (GBOs) formed within 1–2 weeks. Stable sphere growth from tumor fragments confirmed successful GBO establishment. For culture over one month, organoids were cut into 200–500 µm pieces to avoid central necrosis from poor nutrient/oxygen penetration. GBOs underwent histological staining identical to primary tumor tissues.

### 4.17 Neurosphere Assays and ELDA

For neurosphere assays, cells were plated under limiting dilution or defined low‐density conditions and treated as indicated. Neurosphere formation efficiency was calculated as the percentage of wells or seeded cells forming spheres. Stem cell frequency was estimated using extreme limiting dilution analysis (ELDA).

### 4.18 In Vitro DLD Lactylation Assay and Kinetic Analysis

Purified recombinant DLD and AARS2 proteins were used for in vitro lactylation assays. Reactions were performed in a total volume of 30 µL containing 50 mm HEPES (pH 7.5), 25 mm KCl, 2 mm MgCl_2_, 4 mm ATP, 10 nm recombinant AARS2, and 1 µg recombinant DLD per reaction (∼0.62 µm, based on the molecular weight of DLD). L‐lactate was added at the indicated concentrations. Reactions were incubated at 37°C and terminated by heat denaturation.

For time‐course assays, reaction products were collected at 0, 10, 20, 30, 40, 50, and 60 min. DLD K445 lactylation was quantified by dot blot using a DLD‐K445la‐specific antibody. A synthetic lactylated DLD‐K445 peptide corresponding to the modified sequence surrounding K445 was serially diluted to generate a standard curve. Equal volumes of peptide standards and reaction products were spotted onto nitrocellulose membranes, air‐dried, and subjected to immunoblot detection using the DLD‐K445la‐specific antibody. Signal intensities were quantified by densitometric analysis, and the amount of DLD K445 lactylation in each reaction was calculated from the peptide standard curve.

For kinetic analysis, L‐lactate was varied at 0, 0.25, 0.5, 1, 2, 4, 8, and 12 mm, while recombinant AARS2 (10 nm), recombinant DLD (1 µg per reaction; ∼0.62 µm), and ATP (4 mm) were maintained at constant concentrations. Initial reaction velocities (v_0_) were calculated from product formation during the experimentally determined linear phase of the reaction (0‐20 min). Initial velocities were plotted against L‐lactate concentration and fitted to the Michaelis‐Menten equation by nonlinear regression using GraphPad Prism to determine Km and Vmax. The turnover number (kcat) was calculated as kcat = Vmax/[E], where [E] represents the molar concentration of recombinant AARS2 (10 nm) used in the reaction. Catalytic efficiency was calculated as kcat/Km. All kinetic experiments were performed in three independent replicates.

WT DLD and K445R mutant DLD were subjected to identical in vitro lactylation conditions to evaluate the contribution of K445 to AARS2‐dependent DLD lactylation.

### 4.19 DLD Intrinsic Enzymatic Activity Assay

The intrinsic enzymatic activity of recombinant DLD was determined spectrophotometrically by monitoring NADH formation at 340 nm. Recombinant WT DLD, lactylated WT DLD, and K445R mutant DLD were assayed under identical conditions. The reaction mixture (300 µL final volume) contained 50 mM potassium phosphate (pH 7.3), 165 µM NAD^+^, 0.9 mM dihydrolipoic acid (DHLA), and recombinant human DLD at a final concentration of 0.5 µg/mL. Following pre‐incubation at 37°C for 15 min, the reaction was initiated by addition of DHLA. The increase in absorbance at 340 nm was continuously monitored at 37°C using a 96‐well plate reader. Initial reaction rates were calculated from the linear increase in absorbance at 340 nm between 2 and 6 min. NADH formation was calculated using the molar extinction coefficient of NADH determined under the assay conditions. One unit (U) of DLD activity was defined as the amount of enzyme catalyzing the formation of 1 µmol NADH per minute, and specific activity was expressed as U/mg protein. The lactylation status of the recombinant proteins was verified by immunoblotting before enzymatic measurement. All experiments were performed in three independent replicates.

### 4.20 Peptide Pull‐Down, Competition, and Interaction Assays

Biotinylated K445‐pe or K445R‐pe peptides were used for peptide pull‐down assays. For endogenous protein binding assays, MES505 cell lysates were incubated with biotinylated peptides, followed by capture with streptavidin beads. After extensive washing, peptide‐associated proteins were eluted and analyzed by immunoblotting for DLD and AARS2.

For competition assays, cell lysates were incubated with biotinylated K445‐pe in the presence of increasing concentrations of unlabeled K445‐pe or K445R‐pe peptides. The specificity of peptide binding was evaluated by detecting DLD enrichment in streptavidin pull‐down fractions.

To assess direct interaction between K445‐pe and DLD, biotinylated peptides were incubated with purified recombinant DLD protein, and peptide‐bound DLD was detected by immunoblotting. To examine the effect of K445‐pe on the AARS2‐DLD interaction, MES505 cells were treated with K445‐pe or K445R‐pe, followed by immunoprecipitation of endogenous DLD. The associated AARS2 protein was analyzed by immunoblotting.

### 4.21 Immunoprecipitation (IP), GST Pull‐Down Assay, and Western blot

Cells were lysed with EBC lysis buffer containing protease and phosphatase inhibitors. Whole‐cell lysate (WCL) protein concentrations were quantified via BCA assay. Equal protein loads were resolved by 10% SDS–PAGE and transferred to PVDF membranes for Western blot. Membranes were probed with primary antibodies at 4°C overnight, then with HRP‐secondary antibodies at room temperature for 1 h; bands were visualized by ECL. For co‐IP: WCL were incubated with target antibodies at 4°C for 3–4 h, followed by Protein A/G beads overnight. For GST pull‐down: lysates were incubated with glutathione Sepharose beads for 2 h at 4°C. All immunocomplexes were washed 3 times with NETN buffer, separated by SDS–PAGE and immunoblotted.

### 4.22 Peptide Synthesis

All peptides were synthesized by Guoping Pharmaceutical Inc. (Hefei, China) via solid‐phase synthesis and HPLC‐purified to > 98% purity for in vitro and in vivo tests. Peptides for in vivo use consist of all D‐amino acids. Lyophilized peptides were dissolved in PBS as 10 mm stock for cell experiments. For animal assays, K445‐pe or control K445R‐pe was dissolved in sterile PBS to a final concentration of 1 mg/mL (corresponding to an in vivo dose of 10 mg/kg body weight). Mice received tail vein intravenous injections at a fixed volume of 10 µL per gram of body weight every 2 days throughout the treatment cycle.

### 4.23 Immunohistochemistry Staining and Evaluation of Tissue Microarray

Formalin‐fixed, paraffin‐embedded glioblastoma tissue microarray sections were obtained from Harbin Medical University and subjected to immunohistochemical staining. Immunohistochemical staining for DLD‐K445 lactylation was performed by Servicebio (Wuhan, China) according to the manufacturer's standard protocols. Briefly, tissue sections were deparaffinized, rehydrated, and subjected to antigen retrieval. After blocking endogenous peroxidase activity and nonspecific binding, sections were incubated with primary antibodies against DLD‐K445la overnight at 4 °C, followed by incubation with secondary antibodies and visualization using a DAB chromogenic detection system. Slides were counterstained with hematoxylin, dehydrated, and mounted for imaging.

Immunohistochemical staining was independently evaluated by two blinded investigators. The proportion of DLD‐K445la‐positive tumor cells was scored as 0 (0%), 1 (1–25%), 2 (26–50%), 3 (51–75%), or 4 (> 75%), and staining intensity was scored as 0 (negative), 1 (weak), 2 (moderate), or 3 (strong). The IHC score was calculated by multiplying the proportion score by the staining intensity score, yielding a total score ranging from 0 to 12. Based on the distribution of DLD‐K445la staining scores in the cohort, an IHC score of 6 was predefined as the cutoff before survival analysis. Tumors with IHC scores ≤ 6 were classified as DLD‐K445la‐low, whereas those with scores > 6 were classified as DLD‐K445la‐high. The same cutoff was applied to the Kaplan‐Meier analyses of overall survival and progression‐free survival, with differences between groups assessed using log‐rank tests.

### 4.24 Quantification and Statistical Analysis

All quantitative data are presented as the mean ± standard deviation (SD), unless otherwise indicated. Unless otherwise specified, in vitro experiments were performed using at least three biologically independent replicates. Biological replicates represent independently cultured and experimentally treated samples, whereas technical replicates represent repeated measurements of the same biological sample and were not treated as independent observations for statistical testing. The number of biological replicates (n) is indicated in the corresponding figure legends. Comparisons between two groups were performed using two‐sided unpaired Student's t‐tests, whereas comparisons among multiple groups were performed using one‐way or two‐way analysis of variance (ANOVA), as appropriate, followed by the multiple‐comparison tests specified in the corresponding figure legends. Dose‐response curves were analyzed by nonlinear regression, with curve comparisons performed using the extra sum‐of‐squares F test where indicated. Extreme limiting dilution assays were analyzed using ELDA and the χ^2^ test. Survival curves were estimated using the Kaplan–Meier method and compared using the log‐rank (Mantel–Cox) test. Statistical analyses were performed using GraphPad Prism 10 unless otherwise indicated. A two‐sided *p* < 0.05 was considered statistically significant.

### 4.25 Public Human Glioma Datasets

Public glioma transcriptomic datasets were acquired from TCGA, while proteomic data were obtained from the CPTAC cohort.

## Author Contributions

H.W., M.D., H.X., C.L., and S.W. conceived the study and designed the experiments. M.D., H.X., J.Y., T.F., Y.D., X.J., and Y.C. performed the experiments and collected the data. M.D., H.X., H.L., R.L., and Z.C. analyzed and interpreted the data. M.D., H.X., and H.W. drafted the manuscript. H.W., H.L., R.L., and Z.C. revised the manuscript. H.W. supervised the study and acquired funding. All authors reviewed and approved the final manuscript.

## Funding

This work was supported by grants from the National Natural Science Foundation of China (82573129 and 82273164 to H.W.) and the Major Basic Research Fund of Jiangsu Province Hospital (TS202405 to H.W.).

## Ethics Statement

Tumor sample collection was approved by the Institutional Review Board of the First Affiliated Hospital with Nanjing Medical University(2025‐SRFA‐068), with written informed consent from all patients. This study involved animal experiments that were approved by the Ethics Review Committee of Nanjing Medical University (IACUC‐2411056).

## Conflicts of Interest

The authors declare no conflicts of interest.

## Supporting information




**Supporting File 1**: advs78046‐sup‐0001‐SuppMat.docx.

**Supporting File 2**: advs78046‐sup‐0002‐TableS1.xlsx.

**Supporting File 3**: advs78046‐sup‐0003‐TableS2.xlsx.

## Data Availability

The data that support the findings of this study are available within the article and its supplement.
