# Supplementary material for: AARS2‐SIRT5‐Driven DLD K445 Lactylation Promotes Cuproptosis Resistance in Glioblastoma Stem Cells
Source: Adv Sci (Weinh). 2026 Sep 29:e78046. Online ahead of print. doi: 10.1002/advs.78046 (PMC13621201; doi:10.1002/advs.78046)
Supplement: Supplementary file 1 [file ADVS-9999-e78046-s001.docx]

**Supplementary Information**

**AARS2-SIRT5-Driven DLD K445 Lactylation Promotes Cuproptosis Resistance in Glioblastoma Stem Cells**

**Mingtian Ding, Haibiao Xu, Cen Liu, Yangyin Ding, Shuai Wang, Jun Yin, Tao Fu, Xiao Jiang, Yuning Chen, Hui Luo, Rui Li, Zhengxin Chen, Huibo Wang**

**Supplementary Fig. S1. Single-cell clustering and cell-type annotation of glioblastoma samples, related to Figure 1.**
**A** UMAP visualization of glioblastoma single-cell transcriptomes colored by unsupervised cluster identity. **B** UMAP visualization colored by sample origin. **C** Relative abundance of annotated cell populations across individual samples. **D** Feature plots showing representative canonical marker genes used to assign cell-type identities. **E** Heatmap showing scaled expression of selected marker genes across annotated cell populations. No statistical test was performed for this annotation analysis.

**Supplementary Fig. S2. Lactate metabolism is associated with cuproptosis resistance and ES-Cu response, related to Figure 1.**
**A** UMAP visualization of LactateScore across glioblastoma single cells. **B** LactateScore in malignant and non-malignant cell populations. **C** Correlation between LactateScore and CuproResScore at the patient level in malignant cells. **D** CuproResScore in LactateScore-low and LactateScore-high tumors from the CPTAC and TCGA cohorts. **E** Representative immunofluorescence images showing LDHA (yellow) and ALDH1A3 (red) expression in human GBM specimens, together with quantification of the proportion of LDHA-positive cells within the ALDH1A3-positive population. Quantification was performed using nine randomly selected fields from three independent tumors. Scale bar, 50 μm. **F** Representative immunofluorescence images showing DLAT (yellow) and ALDH1A3 (red) expression in human GBM specimens, together with quantification of the proportion of DLAT-positive cells within the ALDH1A3-positive population. Quantification was performed using nine randomly selected fields from three independent tumors. Scale bar, 50 μm. Statistical significance was assessed using the Wilcoxon rank-sum test (B and D), Spearman correlation analysis (C).

**Supplementary Fig. S3. Manipulation of lactate metabolism alters ES-Cu sensitivity in mesenchymal-like GSCs, related to Figure 1.**

**A** Sensitivity of established glioma cell lines and patient-derived GSC models to ES-Cu treatment, shown as dose–response curves and IC50 values. **B–D** Effects of LDH inhibition on protein lactylation and intracellular lactate levels in MES505 and MES21 cells. **E,F** Cell viability of MES505 and MES21 cells treated with ES-Cu in the presence or absence of LDH inhibitor. **G,H** Neurosphere formation and limiting dilution analyses of MES21 cells treated with vehicle or LDH inhibitor in the presence or absence of ES-Cu. **I–K** Effects of NALA supplementation on protein lactylation and intracellular lactate levels in MES505 and MES21 cells. **L,M** Cell viability of MES505 and MES21 cells treated with ES-Cu in the presence or absence of NALA. **N,O** Neurosphere formation and limiting dilution analyses of MES21 cells treated with vehicle or NALA in the presence or absence of ES-Cu. **P** Immunoblot validation of LDHA knockdown in MES505 and MES21 cells. **Q,S** Intracellular lactate levels following LDHA depletion in MES505 and MES21 cells. **R,T** Sensitivity of LDHA-depleted MES505 and MES21 cells to ES-Cu treatment. Data are presented as mean ± SD. n = 3 biologically independent experiments unless otherwise indicated. Statistical significance was assessed using one-way ANOVA followed by Tukey’s multiple-comparison test (C, D, J, K, Q and S), two-way ANOVA followed by Tukey’s multiple-comparison test (E, F, G, N, L, M, R and T), and ELDA χ² analysis (H and O). Original unprocessed blot images are provided in Supplementary Figure S12G.

**Supplementary Fig. S4. Lactate-dependent regulation and validation of DLD lactylation, related to Figure 2.**
**A** Expression of cuproptosis-associated genes following treatment with NALA or L-lactate. **B** Intracellular lactate levels in HEK293T cells treated with increasing concentrations of MCT1/4 inhibitor. **C,D** Effects of MCT1/4 inhibition (C) or GLO1 inhibition (D) on HA-DLD lactylation detected by pan-lactylation immunoblotting following HA-DLD immunoprecipitation. **E** Time-dependent in vitro lactylation of recombinant DLD mediated by AARS2 in the presence of lactate and ATP. DLD-K445la levels were detected by immunoblotting. **F** DLD-K445 lactylation analysis of HA-DLD WT and K445R mutant proteins. **G** Sequence alignment showing conservation of the DLD K445 residue across different species. **H–J** Representative MS/MS spectra identifying additional DLD lactylation sites at K267 (H), K430 (I), and K159 (J). **K** Dot-blot validation of the DLD-K445la antibody using synthetic lactylated and non-lactylated DLD K445 peptides. **L** Ubiquitination analysis of HA-DLD WT and K445R proteins following MG132 treatment.

**M** Cycloheximide-chase analysis showing the stability of HA-DLD WT and K445R proteins in HEK293T cells. Data are presented as mean ± SD. n = 3 biologically independent experiments where indicated. Statistical significance was assessed using the appropriate statistical tests described in the Methods. Representative immunoblots, dot blots, and MS/MS spectra are shown. NS, not significant. Original unprocessed blot images are provided in Supplementary Figure S12H.

**Supplementary Fig. S5. Three-dimensional ribbon structure of human dihydrolipoamide dehydrogenase (E3, DLD) homodimer with lactylation modification at the catalytic lysine site (structural template: PDB ID 2F5Z), related to Figure 3.**

**A** The two identical subunits of the DLD/E3 homodimer are colored yellow and magenta, respectively, in ribbon representation. Covalently bound flavin adenine dinucleotide cofactor (FAD-480) in each catalytic pocket is shown as stick model. The residue labeled Lys-410 in this crystal structure corresponds to Lys-445 in full-length DLD, as the 2F5Z template lacks the first 35 N-terminal amino acids cleaved during DLD protein maturation. Lactylation modification was artificially introduced at this conserved catalytic lysine residue for subsequent molecular simulation, with the modified sites highlighted and labeled in both monomers. The initial crystal coordinate file was retrieved from the RCSB PDB database (https://www.rcsb.org/structure/2F5Z), and post-translational lysine lactylation modification was reconstructed on the truncated template structure. **B–F** Molecular dynamics analyses of the PDH complex containing WT or K445-lactylated DLD throughout the simulation：**B** Radius of gyration (Rg). **C** Root-mean-square deviation (RMSD). **D** Root-mean-square fluctuation (RMSF). **E** Solvent-accessible surface area (SASA). **F** Hydrogen-bond number. No statistical analysis was applied to molecular dynamics trajectories.

**Supplementary Fig. S6. DLD K445 lactylation regulates PDH activity, DLAT lipoylation, and metabolic reprogramming, related to Figure 3.**
**A** PDH activity in MES21 cells following treatment with LDH inhibitor and/or NALA. **B** Immunoblot validation of DLD knockdown and re-expression of HA-DLD WT or K445R in MES505 and MES21 cells. **C** PDH activity in MES21 cells expressing HA-DLD WT or K445R in the presence or absence of NALA. **D** PDH activity in MES21 cells expressing HA-DLD WT or K445R following AARS2 knockdown. **E–H** Effects of AARS2 depletion, DLD depletion, NALA treatment, or LDH inhibition on PDH activity in MES505 and MES21 cells. **I** DLAT lipoylation in MES21 cells treated with NALA and/or LDH inhibitor. **J** DLAT lipoylation following DLD depletion combined with LDH inhibition in MES505 and MES21 cells. **K** DLAT lipoylation following AARS2 depletion combined with NALA treatment in MES505 and MES21 cells. **L,M** Oxygen consumption rate (OCR) profiles and quantification of basal and maximal respiration in MES21 cells expressing DLD WT or K445R. **N,O** Extracellular acidification rate (ECAR) profiles and quantification of glycolytic rate and glycolytic capacity in MES21 cells expressing DLD WT or K445R.	Data are presented as mean ± SD. n = 3 biologically independent samples for A, C–H, M, and O. Statistical significance was assessed using one-way ANOVA followed by Tukey’s multiple-comparison test (A and C–H) and two-sided unpaired t test (M and O). Representative immunoblots are shown. Original unprocessed blot images are provided in Supplementary Figure S12I.

**Supplementary Fig. S7. Metabolic inhibition and DLD K445R enhance ES-Cu-induced cuproptosis features, related to Figure 4.**
**A,B** Representative DLAT immunofluorescence images and quantification of DLAT puncta in MES505 cells treated with NALA or LDH inhibitor in the presence or absence of ES-Cu. Scale bars, 10 μm. **C** DLAT oligomer formation in MES505 cells exposed to increasing concentrations of ES-Cu under vehicle, NALA, or LDH inhibitor treatment. **D,E** Representative DLAT immunofluorescence images and quantification of DLAT puncta in MES21 cells treated as described in A and B.Scale bars, 10 μm. **F** DLAT oligomer formation in MES21 cells exposed to increasing concentrations of ES-Cu under vehicle, NALA, or LDH inhibitor treatment. **G** ES-Cu dose-response curves in MES21 cells expressing DLD WT or K445R. **H,I** Neurosphere formation and limiting dilution analyses of MES21 cells expressing DLD WT or K445R in the presence or absence of ES-Cu. **J** Expression of stemness-associated markers in MES21 cells expressing HA-DLD WT or K445R following ES-Cu treatment. **K,L** Representative bioluminescence images and quantification of intracranial MES21 tumors expressing DLD WT or K445R with or without ES-Cu treatment. **M** Immunohistochemical staining of DLD-K445la, DLAT, and lipoic acid in tumor sections from the indicated groups. Scale bars, 200 μm. **N** Kaplan–Meier survival analysis of mice bearing MES21-derived tumors expressing DLD WT or K445R and treated with or without ES-Cu. Data are presented as mean ± SD. n = 3 biologically independent samples for B, E, and G; n = 5 independent sphere cultures for H; n is indicated by individual data points for L. Statistical significance was assessed using two-way ANOVA followed by Tukey’s multiple-comparison test (B, E, H, and L), nonlinear regression with extra sum-of-squares F test (G), ELDA χ² analysis (I), and log-rank (Mantel–Cox) test (N). Original unprocessed blot images are provided in Supplementary Figure S12J.

**Supplementary Fig. S8. SIRT5-mediated DLD delactylation regulates ES-Cu sensitivity, related to Figure 5.**
**A,B** DLD K445 lactylation in HA-DLD immunoprecipitates from MES21 cells following NAM or NMN treatment. **C** MS/MS spectrum identifying the SIRT5-derived peptide FHFQGPCGTTLPEALACHENETVS. **D,E** Reciprocal co-immunoprecipitation analyses demonstrating endogenous interaction between DLD and SIRT5 in MES21 cells. **F,G** Relative PDH activity following expression of Flag-SIRT5 WT or H158Y, or after SIRT5 knockdown in MES21 cells. **H,I** Intracellular lactate levels following SIRT5 depletion in MES21 cells. **J** Relative PDH activity in MES21 cells expressing Flag-SIRT5 in the presence or absence of AARS2 knockdown. **K,L** DLAT lipoylation following Flag-SIRT5 expression combined with AARS2 or DLD depletion in MES21 cells. **M** ES-Cu dose-response curves of MES21 cells expressing Flag-SIRT5 and/or shAARS2. **N,O** Representative bioluminescence images and quantification of intracranial MES21 tumors from the indicated groups. **P,Q** Kaplan–Meier survival analyses of mice bearing intracranial MES505- or MES21-derived tumors subjected to the indicated treatments. Data are presented as mean ± SD. n = 3 biologically independent samples for F–J and M; n is indicated by individual data points in O. Statistical significance was assessed using one-way ANOVA followed by Tukey’s multiple-comparison test (F–J and O), nonlinear regression with extra sum-of-squares F test (M), and log-rank (Mantel–Cox) test (P and Q). NS, not significant. Original unprocessed blot images are provided in Supplementary Figure S12K.

**Supplementary Fig. S9. Validation of the specificity of the DLD K445 lactylation-targeting peptide, related to Figure 7.**

**A** Biotin-K445-pe pull-down assay from MES505 cell lysates showing enrichment of DLD and weaker co-pull-down of AARS2 by K445-pe compared with the Biotin-K445R-pe control. **B** Competition assay using increasing amounts of unlabeled K445-pe or K445R-pe peptides to evaluate the sequence specificity of K445-pe binding to DLD. **C** Direct binding assay of K445-pe with purified recombinant DLD showing that Biotin-K445-pe, but not Biotin-K445R-pe, directly pulls down DLD. **D** Co-immunoprecipitation analysis showing that K445-pe disrupts the interaction between DLD and AARS2, whereas K445R-pe has no obvious effect. Representative immunoblots are shown. Original unprocessed blot images are provided in Supplementary Figure S12L.

**Supplementary Fig. S10. Optimization and functional validation of DLD K445-targeting peptides, related to Figure 7.**
**A** Amino acid sequences of the candidate peptides designed to target the DLD K445 region. **B** Screening of candidate peptides for their ability to inhibit DLD K445 lactylation in HA-DLD immunoprecipitates. **C,D** Effects of increasing concentrations of K445-pe or K445R-pe on DLD K445 lactylation in HEK293T cells. **E** DLAT lipoylation in MES21 cells following treatment with K445-pe or K445R-pe. **F,G** Representative images and quantification of DLAT puncta in MES21 cells treated with vehicle, K445-pe, or K445R-pe in the presence or absence of ES-Cu. Scale bars, 10 μm. **H** DLAT oligomerization in MES21 cells exposed to increasing concentrations of ES-Cu with vehicle, K445-pe, or K445R-pe treatment. **I** ES-Cu dose-response curves of MES21 cells treated with vehicle, K445-pe, or K445R-pe. **J,K** Neurosphere formation and limiting dilution analyses of MES21 cells treated with K445-pe or K445R-pe, with or without ES-Cu. **L** Expression of mesenchymal stemness-associated markers in MES21 cells subjected to the indicated treatments. **M,N** Representative images and growth quantification of patient-derived glioblastoma organoids treated with vehicle, K445-pe, or K445R-pe. **O,P** Representative images and growth quantification of patient-derived glioblastoma organoids treated with ES-Cu alone or in combination with K445-pe or K445R-pe. Data are presented as mean ± SD. n = 3 biologically independent samples for G, I, J, N, and P. Statistical significance was assessed using two-way ANOVA followed by Tukey’s multiple-comparison test (G and J), nonlinear regression with extra sum-of-squares F test (I), ELDA chi-square test (K), and two-way repeated-measures ANOVA followed by Tukey’s multiple-comparison test (N and P). NS, not significant. Original unprocessed blot images are provided in Supplementary Figure S12M.

**Supplementary Fig. S11. Synergistic antitumor activity and safety assessment of K445-pe–based combination therapy, related to Figure 7.**
**A–D** Quantification of DLD-K445la, DLAT, lipoic acid, and Ki67 staining in tumors from the indicated treatment groups. **E–H** Response-surface analyses and synergy heatmaps showing the combined effects of ES-Cu and K445-pe in MES505 and MES21 cells. **I,J** Representative H&E-stained sections of brain, liver, kidney, heart, and lung tissues from mice treated with vehicle, K445-pe, or K445R-pe, in the absence or presence of ES-Cu. Scale bars, 2 mm (brain panels); 100 μm (liver, kidney, heart and lung panels). **K–R** Serum biochemical parameters, including total protein (TP), albumin (ALB), total bilirubin (TBIL), alkaline phosphatase (ALP), alanine aminotransferase (ALT), aspartate aminotransferase (AST), creatinine (CREA), and urea (UREA), measured in mice from the indicated treatment groups. Data are presented as mean ± SD. n is indicated by individual data points. Statistical significance was assessed using one-way ANOVA followed by Tukey’s multiple-comparison test (A–D and K–R). NS, not significant.

**Supplementary Fig. S12. Original unprocessed blot images.** Original unprocessed images corresponding to blot-based experiments included in the study. Panels A–F correspond to Figures 2–7, respectively; panels G–M correspond to Supplementary Figures S3, S4, S6, S7, S8, S9, and S10, respectively. Red boxes indicate the regions displayed in the corresponding figures. Molecular-weight markers and target-protein labels are retained where available.

Supplementary Figure S12A. Original unprocessed blots for Figure 2

Supplementary Figure S12A. Original unprocessed blots for Figure 2

Supplementary Figure S12A. Original unprocessed blots for Figure 2

Supplementary Figure S12B. Original unprocessed blots for Figure 3

Supplementary Figure S12B. Original unprocessed blots for Figure 3

Supplementary Figure S12B. Original unprocessed blots for Figure 3

Supplementary Figure S12C. Original unprocessed blots for Figure 4

Supplementary Figure S12D. Original unprocessed blots for Figure 5

Supplementary Figure S12D. Original unprocessed blots for Figure 5

Supplementary Figure S12D. Original unprocessed blots for Figure 5

Supplementary Figure S12E. Original unprocessed blots for Figure 6

Supplementary Figure S12F. Original unprocessed blots for Figure 7

Supplementary Figure S12F. Original unprocessed blots for Figure 7

Supplementary Figure S12G. Original unprocessed blots for Supplementary Figure S3

Supplementary Figure S12H. Original unprocessed blots for Supplementary Figure S4

Supplementary Figure S12H. Original unprocessed blots for Supplementary Figure S4

Supplementary Figure S12I. Original unprocessed blots for Supplementary Figure S6

Supplementary Figure S12I. Original unprocessed blots for Supplementary Figure S6

Supplementary Figure S12I. Original unprocessed blots for Supplementary Figure S6

Supplementary Figure S12J. Original unprocessed blots for Supplementary Figure S7

Supplementary Figure S12K. Original unprocessed blots for Supplementary Figure S8

Supplementary Figure S12K. Original unprocessed blots for Supplementary Figure S8

Supplementary Figure S12L. Original unprocessed blots for Supplementary Figure S9

Supplementary Figure S12M. Original unprocessed blots for Supplementary Figure S10

Supplementary Figure S12M. Original unprocessed blots for Supplementary Figure S10
